# Direct Cyclopalladation of Fluorinated Benzyl Amines by Pd_3_(OAc)_6_: The Coexistence of Multinuclear Pd_*n*_ Reaction Pathways Highlights the Importance of Pd Speciation in C–H Bond Activation

**DOI:** 10.1021/acs.organomet.3c00178

**Published:** 2023-05-31

**Authors:** Ian J. S. Fairlamb, Jan Lang, Aleš Růžička, Miloš Sedlák, Jiří Váňa

**Affiliations:** † Department of Chemistry, University of York, Heslington, York YO10 5DD, United Kingdom; ‡ Department of Low Temperature Physics, Faculty of Mathematics and Physics, Charles University, V Holešovičkách 747/2, 18000 Prague 8, Czech Republic; § Department of General and Inorganic Chemistry, Faculty of Chemical Technology, University of Pardubice, Studentská 573, 53210 Pardubice, Czech Republic; ∥ Faculty of Chemical Technology, Institute of Organic Chemistry and Technology, University of Pardubice, Studentská 573, 53210 Pardubice, Czech Republic

## Abstract

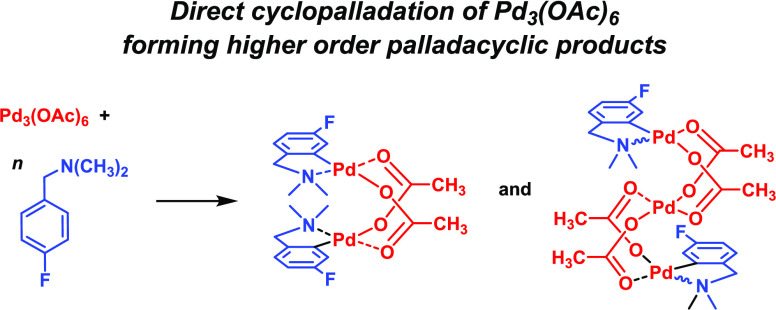

Palladacycles are key intermediates in catalytic C–H bond functionalization reactions and important precatalysts for cross-couplings. It is commonly believed that palladacycle formation occurs through the reaction of a substrate bearing a C–H bond *ortho* to a suitable metal-directing group for interaction with, typically, mononuclear “Pd(OAc)_2_” species, with cyclopalladation liberating acetic acid as the side product. In this study, we show that *N*,*N*-dimethyl-fluoro-benzyl amines, which can be cyclopalladated either *ortho* or *para* to fluorine affording two regioisomeric products, can occur by a direct reaction of Pd_3_(OAc)_6_, proceeding via higher-order cyclopalladated intermediates. Regioselectivity is altered subtly depending on the ratio of substrate:Pd_3_(OAc)_6_ and the solvent used. Our findings are important when considering mechanisms of Pd-mediated reactions involving the intermediacy of palladacycles, of particular relevance in catalytic C–H bond functionalization chemistry.

## Introduction

**Figure 1:**
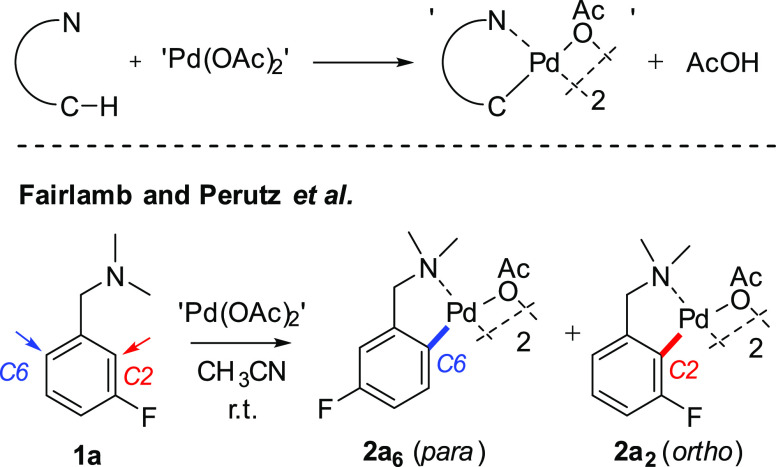
Top: cyclopalladation of suitable substrates bearing a metal-directing group (depicted here with nitrogen); “Pd(OAc)_2_” can be derived from different palladium(II) diacetate sources, *e.g.*, Pd_3_(OAc)_6_ or [Pd_2_(OAc)_4_]_*n*_ (less commonly). Bottom: cyclopalladation of **1a** to give regioisomeric products **2a_6_** (*para*) and **2a_2_** (*ortho*), as reported by Fairlamb and Perutz *et al.* (note that the palladacyclic numbering system is that used in the paper).^10^

The cyclopalladation of substrates bearing a suitable metal-directing group and a proximal C–H bond, *e.g.*, N^CH, at “Pd(OAc)_2_” is a commonly encountered reaction in both the synthesis of palladacycles and catalytic C–H functionalization processes (Figure 1).^1^ Moreover, such palladacycles are invoked as key intermediates, either on- or off-cycle, for many different types of reactions.^2^ Palladacyclic products can be mononuclear, dinuclear, or trinuclear (*vide supra*). In most studies, cyclopalladation is thought to occur via a mononuclear Pd^II^ species (Figure 1), with the general presumption that despite the form of the “Pd(OAc)_2_” starting material used, *i.e.*, Pd_3_(OAc)_6_ or polymeric [Pd_2_(OAc)_4_]_*n*_^3^ species, mononuclear “Pd(OAc)_2_” is delivered in solution for direct reaction with the N^CH substrate. However, the occurrence of palladacyclic products in dinuclear or trinuclear forms raises the question of whether C–H bond activation takes place through polynuclear Pd species, or if the mononuclear Pd complexes aggregate by alternative mechanisms (the latter being the current state of thinking).^4^

Granell *et al.*(5) proposed that primary benzylamines undergo cyclopalladation involving dinuclear Pd^II^ species in a cooperative manner, where the metalation by the first palladium atom is the rate-limiting step. Furthermore, Musaev *et al.* showed that a single monoprotected amino acid (MPAA)-bridged dipalladium core [Pd_2_(MPAA)_1_] is an active catalyst for cyclopalladation of *N*,*N*-dimethylaminomethylferrocene.^6^

Computational studies reported by Musaev and coworkers pointed to the direct reaction of liberated “Pd_2_(OAc)_4_” from Pd_3_(OAc)_6_, which is arguably the most common commercially used palladium(II) acetate source (note: at the time of writing, Pd_3_(OAc)_6_ is the only “Pd(OAc)_2_” source openly available).^3,7^ While thermodynamically uphill in terms of compound isolation, coordination of a suitable N^CH substrate allows formation of stable intermediates providing a competing pathway for cyclopalladation via the dinuclear Pd^II^ “Pd_2_(OAc)_4_” species. Despite this possibility, the consensus is that cyclopalladation proceeds predominantly via mononuclear Pd^II^ intermediates.

On the other hand, Yu *et al.*(8) invoked reactions of oxazolines with Pd_3_(OAc)_6_ to give a trinuclear Pd^II^ species containing two N-coordinated oxazoline ligands, which undergo monomerization prior to cyclopalladation occurring; formation of mononuclear palladacycles then aggregates to bring about the formation of trinuclear [C^NPd(μ^2^-OAc)_2_Pd(μ^2^-OAc)_2_Pd(C^N)] species, which have been observed in other studies. For example, Váňa *et al.* established that cyclopalladation can occur directly at Pd_3_(TFA)_6_ with acetanilide liberating dinuclear cyclopalladated complexes, “Pd(TFA)_2_”, and carboxylic acid.^9^

The cyclopalladation of a series of fluorinated benzyl amines with Pd_3_(OAc)_6_ was reported by Fairlamb *et al.*(10) For *N*,*N*-dimethyl-3-fluorobenzyl amine **1a** (Figure 1), cyclopalladation was found to be nonselective in CH_3_CN, affording regioisomeric dinuclear [Pd(C^N)(OAc)]_2_ products **2a_6_** and **2a_2_**. Cyclopalladation involving palladium chloride salts, proceeding via a different mechanism, was regioselective placing the fluorine substituent *para* to Pd^II^. A question from that study was whether cyclopalladation of the higher-order Pd_3_(OAc)_6_ cluster might also take place, which was mentioned in the concluding remarks to that study.

In this paper, we report the outcomes of the direct reactions of fluorinated benzyl amines with the cyclic Pd_3_(OAc)_6_ cluster, under varying reaction conditions. Reaction monitoring by NMR spectroscopic analysis shows the formation of different Pd^II^ species, including formation of both trinuclear and dinuclear Pd^II^ species. Our studies indicate that a direct cyclopalladation of *N*,*N*-dimethyl-4-fluorobenzyl amine **1b** with Pd_3_(OAc)_6_ occurred under conditions where there is “excess” Pd over **1b**, resulting in formation of trinuclear clusters of the type [C^NPd(μ^2^-OAc)_2_Pd(μ^2^-OAc)_2_Pd(C^N)]. Furthermore, we reveal a changing regioselectivity in certain substrates.

## Results and Discussion

### Influence of the Solvent

**Figure 2:**
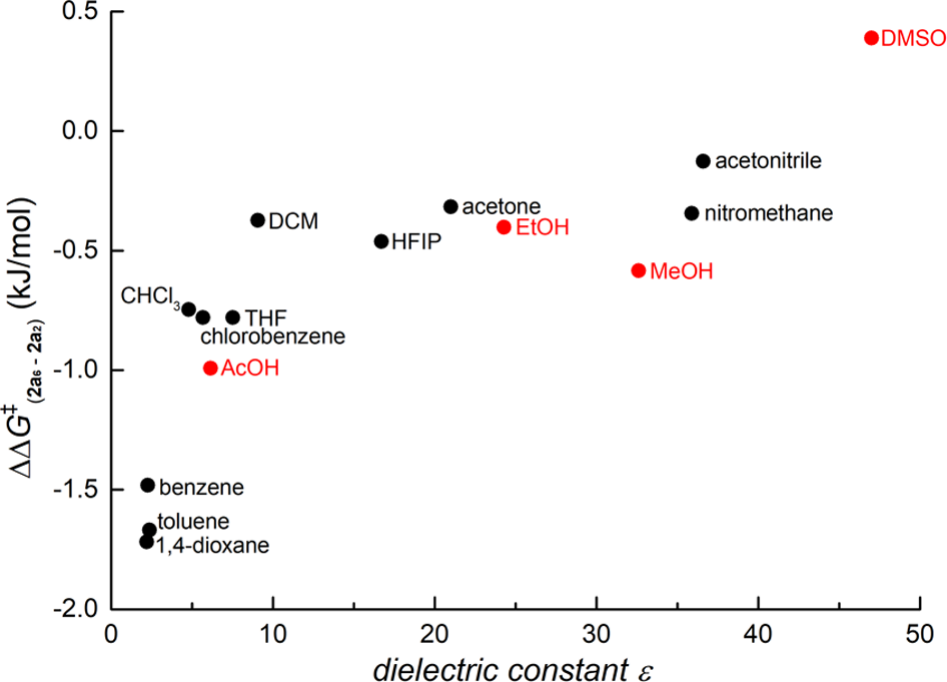
Influence of the solvent on the ratio of regioisomers **2a_6_** and **2a_2_**. Red points are included with some degree of uncertainty. For more details, see Table S1.

We first assessed whether the regioselectivity for cyclopalladation of **1a** with Pd_3_(OAc)_6_ was altered with changing solvent nature (Figure 2 and Table S1, see the Supporting Information). Thus, we conducted the reaction of **1a** (1.3 equiv with respect to Pd) with Pd_3_(OAc)_6_ in different solvents and analyzed (by NMR) the ratio of cyclopalladated products before and after addition of pyridine. The addition of pyridine enables simplification of spectra due to the cleavage of polynuclear complexes and formation of mononuclear complexes of the type *trans*-[C^NPd(OAc)(*N*-pyridine)].^10^ The results displayed in Figure 2 and Table S1 show an increasing amount of the *ortho* activated product **2a_2_** with increasing dielectric constant. It is also pertinent to mention that no temperature effect is observed in two exemplar solvents, namely, toluene and acetonitrile (Table S2, see the Supporting Information).

The reaction outcomes described in Figure 2 can be explained in part by the findings of Lei and coworkers,^11^ who showed that monomerized *trans*-Pd(OAc)_2_(solvent)_2_ is liberated from Pd_3_(OAc)_6_ in more polar solvents (evidenced by *in operando* X-ray absorption spectroscopy).

These data show that solvent polarity lowers the energy for the *ortho*-palladation reaction pathway. In less polar solvents, we expect larger Pd clusters to be dominant, mainly as the solvent is less able to displace the bridging Pd-acetoxy ligands. Thus, the presence of large Pd clusters disfavors the *ortho*-palladation reaction pathway. We cannot rule out changes in character at the nitrogen center influencing transition state structures and geometries.

### Influence of Reaction Stoichiometry

The influence of the reaction stoichiometry on cyclopalladation regioselectivity was next assessed (Table 1). In chloroform (1% EtOH), the ratio of regioisomers **2a_6_** and **2a_2_** remained constant while changing the molar ratio of **1a** versus “Pd(OAc)_2_”. In contrast, in dichloromethane and acetonitrile, changes in the ratio of **2a_6_** and **2a_2_** on lowering the amount of **1a** were observed (Table 1 and Table S3).

**Table 1:** Influence of Reaction Stoichiometry on the Ratio of Regioisomers **2a_6_** and **2a_2_**

	^19^F{^1^H} integral intensities					
solvent	CHCl_3_/1% EtOH		DCM		acetonitrile	
molar ratio of **1a**:“Pd(OAc)_2_”	**2a_6_**	**2a_2_**	**2a_6_**	**2a_2_**	**2a_6_**	**2a_2_**
2.60:1			1	0.93	1	0.99
1.99:1	1	0.76	1	0.89	1	0.95
1.31:1	1	0.74	1	0.85	1	0.95
0.99:1	1	0.72	1	0.83	1	0.92
0.66:1	1	0.72	1	0.80	1	0.87
0.33:1	1	0.73	1	0.77	1	0.85

**Figure 3:**
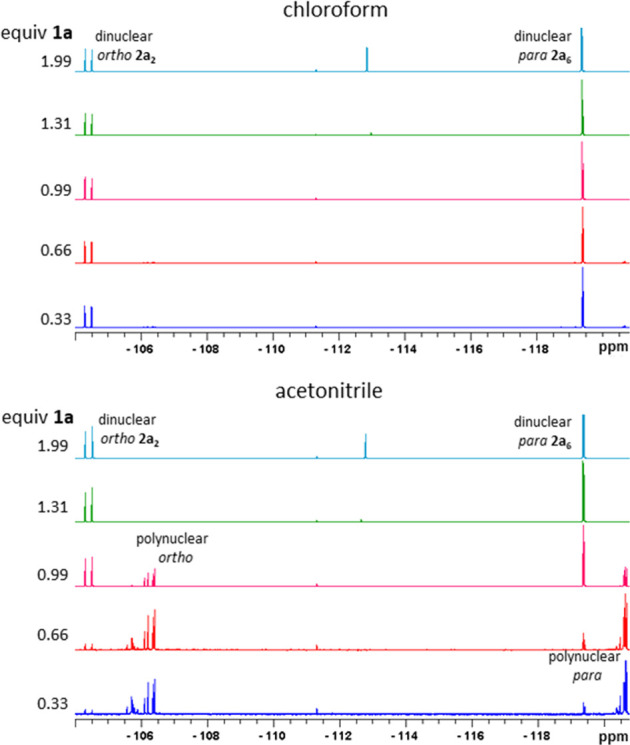
Comparison of ^19^F{^1^H} NMR spectra (measured in CDCl_3_) illustrating product distribution dependence on reaction stoichiometry (different amounts of **1a** added to the one equivalent of “Pd(OAc)_2_”) for experiments running in chloroform (1% EtOH) (top) and acetonitrile (bottom) (for details, see Table S3 and related comments).

The deeper insight into this difference offers comparison of the ^19^F{^1^H} NMR spectra of the individual experiments measured before addition of pyridine (Figure 3). From the spectral data for experiments run in chloroform, only the signals of dinuclear palladacycles **2a_6_** and **2a_2_** are present. However, the experiments in acetonitrile and dichloromethane exhibit a decreasing intensity of signals corresponding to dinuclear palladacycles **2a_6_** and **2a_2_** and increasing intensity of new signals, associated with increasing Pd present in the reaction mixture.

This observation is consistent with the changes observed in Table 1 and can be explained by the opening of a new reaction pathway involving alternative polynuclear reaction intermediates, affording a different ratio of *ortho*- and *para*-substituted palladacycles. This reaction pathway becomes more important with an increasing ratio of palladium(II) acetate in the reaction mixture.

### Kinetic Experiments

**Scheme 1:**
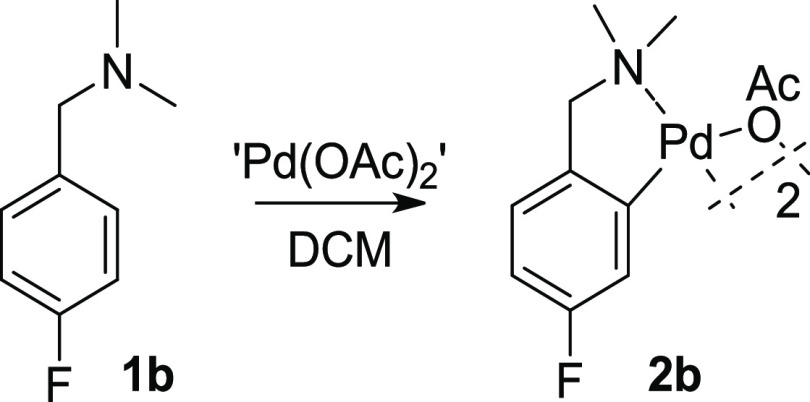
Reaction of **1b** to **2b**

To gain further information about the reaction pathways and intermediates, the kinetic behavior of the system was monitored by ^1^H and ^19^F{^1^H} NMR spectroscopic analysis. To avoid the complicated spectra due to the presence of regioisomeric species, *N*,*N*-dimethyl-4-fluorobenzyl amine **1b** was used as a substrate in the first instance (Scheme 1).

**Figure 4:**
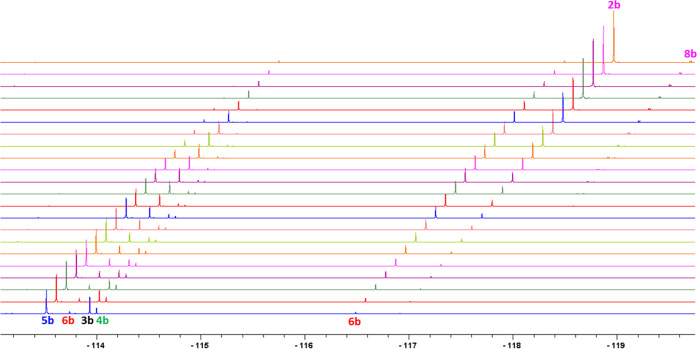
Time-dependent ^19^F{^1^H} NMR spectra of the reaction of a slight excess of **1b** (1.31 equiv) and “Pd(OAc)_2_” (1 equiv) in CD_2_Cl_2_ (0.55 mL) [0.081 M] at room temperature. Time increases from bottom (150 s) to top (7460 s).

The temporal evolution of intermediates and products is visible by ^19^F{^1^H} NMR spectral analysis (Figure 4). Here, the reaction containing a small excess of **1b** (1.3 equiv) over “Pd(OAc)_2_” showed signals of six reaction species (intermediates **3b**, **4b**, **5b**, and **6b** and products **2b** and **8b**) grouped into two spectral regions (Figure 4). The species seen in the region between δ −112 and −114.5 ppm correspond to the signals of fluorine atoms connected to the *N*-coordinated non-C–H activated substrate. On the other hand, in the region of δ −116.5 to −118 ppm are found the fluorine atoms connected to the cyclopalladated species.

**Figure 5:**
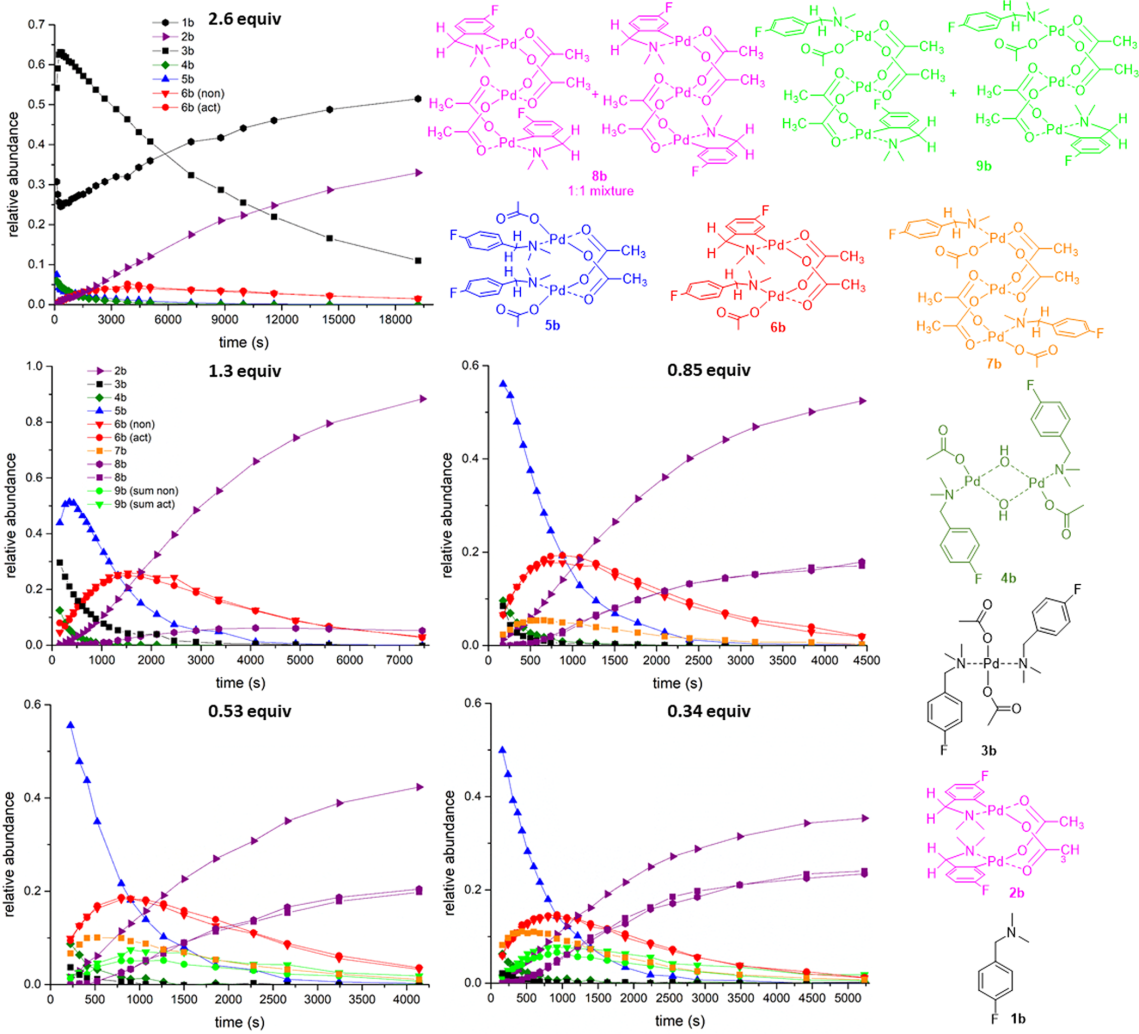
Left: ^19^F signal evolution profiles obtained from ^19^F{^1^H} NMR for reaction of differing quantities of **1b** with “Pd(OAc)_2_” (1 equiv) in CD_2_Cl_2_ at room temperature, where (non)/(act) means nonactivated/activated substrate **1b**. Right: suggested structures of the reaction species.

The product/intermediate evolution profiles obtained by integration of the individual ^19^F{^1^H} NMR signals are depicted in Figure 5. It is shown that each reaction species contains only one type of substrate (likely *N*-coordinated, cyclopalladated). The only exception is **6b**, containing both *N*-coordinated (nonactivated, “non”) and cyclopalladated (activated, “act”) substrate molecules.

The kinetic behavior of solutions containing a different excess of “Pd(OAc)_2_” over **1b** was also examined. Changes were noted in these experiments (Figure 5 and Figures S3–S18, Supporting Information). Firstly, signals corresponding to the second product **8b** and intermediates **7b** and **9b** whose abundance increased with a decreasing **1b**/Pd ratio of reactants appear. While **7b** gives one signal in the region of *N*-coordinated substrates, **9b** gives at least five weak signals in both regions of the spectra, indicative of the presence of cyclopalladated and *N*-coordinated units within the molecule. Secondly, we note that **3b** grows in the presence of an increasing excess of **1b**.

The knowledge gained from the reaction profiles obtained by ^19^F NMR spectroscopic analysis can help to extract analogous ^1^H spectral data, enabling assignment of the acetate ligands to the reaction species (see Figures S3–S7 and S13–S17, Supporting Information). The problematic region is that in the range of 1.8–2.1 ppm in the ^1^H NMR spectra, where there are overlapping signals of N–CH_3_ and acetate CH_3_ groups.

### Signal Assignment

The signals for unreacted **1b** are observable only in the experiment where there is an excess (Figures S7 and S17, see the Supporting Information), as singlets with variable chemical shifts due to an interaction with liberated acetic acid.^12^

The ^19^F signal of the dinuclear product **2b** appears at δ −116.9 ppm. ^1^H NMR exhibits signals for the aromatic protons at δ 6.92 (1H) and 6.75 (2H) ppm. These are accompanied by two “doublets” of diastereotopic benzylic CH_2_ groups at δ 3.65 (1H) and 3.25 (1H) ppm together with two distinctive N–CH_3_ groups at δ 2.82 (3H) and 2.16 (3H, NCH_3_) and a bridging acetate methyl group at δ 2.11 (3H) ppm. These data are characteristic of palladacyclic complexes.^13^

Trinuclear products **8b** give signals at δ −117.60 and −117.62 ppm by ^19^F NMR. The ^1^H NMR spectrum shows signals of six aromatic protons at δ 7.05 (2H), 6.75 (2H), 6,65 (1H), and 6.55 (1H) ppm. Also, four “doublets” of split diastereotopic CH_2_ groups are seen at δ 4.50 (1H), 4.46 (1H), 4.02 (1H), and 3.97 (1H) ppm. Finally, eight signals for acetate CH_3_ and N–CH_3_ groups are seen at δ 3.05 (3H), 3.02 (3H), 3.01 (3H), 2.94 (3H), 2.01 (3H), 1.93 (3H), 1.74 (3H), and 1.67 (3H) ppm. The presence of two sets of signals indicates formation of **8b** in two isomeric forms (as a 1:1 mixture).

We note that the formation of products **2b** and **8b** is accompanied by formation of liberated acetic acid δ 2.15 (3H).

The first two intermediate species **3b** and **4b** give rise to signals for an *N*-coordinated substrate. Furthermore, the benzylic CH_2_ and N–CH_3_ protons are not split, indicating that the substrate units are not bonded tightly, *i.e.*, the complexes are not held in a rigid conformation or alternate configuration. Intermediate **3b** gives one signal at δ −113.9 ppm by ^19^F NMR. The corresponding ^1^H signals at δ 3.66 (2H–CH_2_) and 2.33 (6H–N(CH_3_)_2_) ppm are not coupled to other nuclei. Aromatic protons are found at δ 7.87 (2H) and 7.19 (2H) ppm. The ^1^H signal at δ 1.95 ppm (3H) corresponds to the acetate CH_3_ groups. The abundance of **3b** is increasing with increasing excess of **1b** over Pd. Complex **3b** is most likely mononuclear Pd(OAc)_2_(**1b**)_2_ whose formation is proposed in the first steps of the reaction of Pd_3_(OAc)_6_ with a stoichiometric amount of benzylamine^5^ or a two-fold excess of dimethylbenzylamine.^14^

The fluorine chemical shift of **4b** (^19^F NMR) appears at δ −114 ppm. Signals at δ 8.35 (2H–ArH), 7.35 (2H–ArH), 3.56 (2H–CH_2_), 2.36 (6H), and 1.91 ppm (3H) in ^1^H were also assigned to **4b**. The relative abundance of this intermediate seems to be almost independent of the **1b**/Pd ratio. However, it strongly increases after addition of water to the reaction mixture (Figure S19). We were not able to fully assign its structure and nuclearity; however, it contains Pd/**1b**/OAc in a 1:1:1 ratio; we tentatively suggest that it is a Pd_2_ dimer complex possessing bridging hydroxo ligands.

The next intermediate **5b** contains only an *N*-coordinated substrate, whose ^19^F signal occurs at δ −113.5 ppm. By ^1^H NMR, there are signals indicative of *para* disubstitution at δ 8.25 (2H) and 7.27 (2H) ppm, two “doublets” at δ 4.53 (1H) and 3.72 (1H) ppm corresponding to a diastereotopic CH_2_, and four signals at δ 2.70, 2.22, 2.01, and 1.77 ppm of CH_3_ groups derived from two N–CH_3_ groups (substrate) and two CH_3_ of the bridging acetate ligands at Pd. The splitting of CH_2_ and N(CH_3_)_2_ groups, analogous to the splitting in the dinuclear Pd_2_ product **2b**, suggests an alternative chemical environment. The existence of this intermediate is likely rigid and in a dimeric form (Figure 5).

The intermediate **6b** gives two signals with identical intensities by ^19^F NMR. One signal is in the region of an *N*-coordinated moiety (δ −113.77 ppm) and the second signal in the region for a palladacyclic motif (−116.5 ppm). The ^1^H spectra show the presence of one nonactivated *para*-disubstituted aromatic ring with signals at δ 7.76 (2H) and 7.15 (2H) ppm, along with one activated aromatic ring with signals of three protons at δ 7.05 (1H), 6.85 (1H), and 6.7 (1H) ppm. Furthermore, there are four “doublets” that appear at δ 4.7 (1H), 4.2 (1H), 3.7 (1H), and 3.4 (1H) ppm covering CH_2_ hydrogens. Lastly, seven unique CH_3_ groups that span four different N–CH_3_ environments and three acetates are observed (^1^H signals at δ 3.07, 2.88, 2.05, 2.0, 1.96, 1.94, and 1.88 ppm). These data suggest **6b** to be a dinuclear Pd complex containing one molecule of the nonactivated substrate and one molecule of the activated substrate (Figure 5).

If the reaction occurs with excess “Pd(OAc)_2_”, signals of new but minor intermediates **7b** and **9b**, together with two signals of products **8b**, appear (Figure 3). Their occurrence is concominant with increasing the Pd:**1b** ratio. Unfortunately, due to their lower abundance and overlap with other signals, assignment of all the signals was not possible by ^19^F NMR spectroscopic analysis; structural deductions were thus made by comparison with dinuclear Pd_2_ species.

Complex **7b** provides only one signal for the *N*-coordinated species at δ −112.6 ppm (^19^F NMR). Signals at δ 7.39 (2H–Ar), 4.40 (1H), and 2.78 (3H) ppm were identified by ^1^H NMR spectroscopic analysis. These data taken together point to this being a trinuclear analogue of **5b**.

Finally, **9b** exhibits two fluorine signals at δ −113.77 and −113.82 in the region of *N*-coordinated substrates and three signals at δ −117.34, −117.5, and −117.69 ppm in the region showing cyclopalladated substrates by ^19^F NMR spectroscopic analysis. This is most likely a trinuclear Pd_3_ analogue of **6b**.

**Figure 6:**
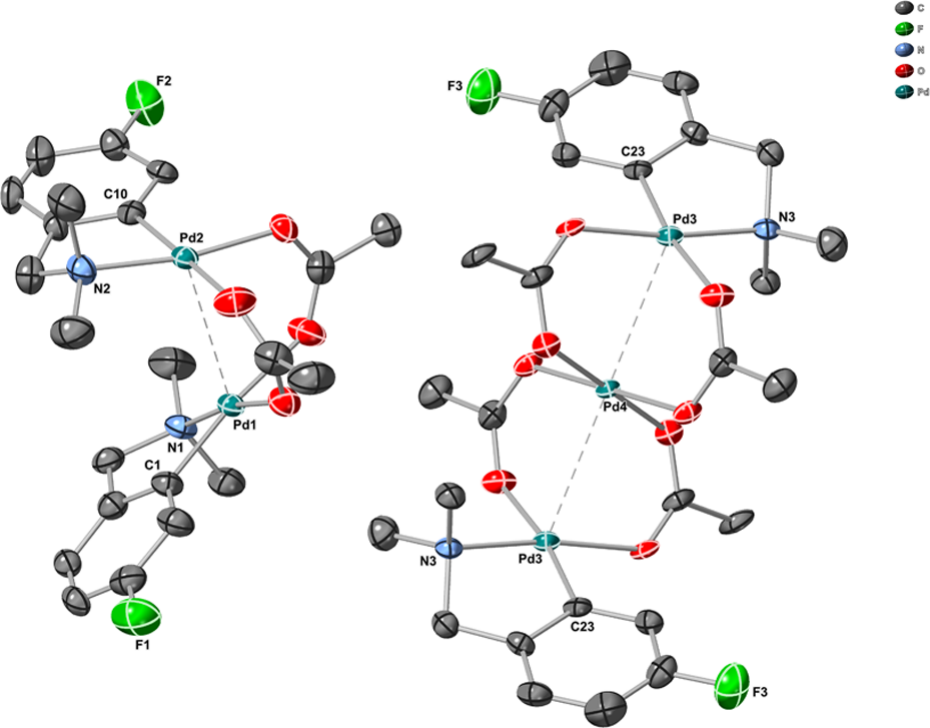
X-ray structure of a cocrystal of **2b** and **8b** (image produced from the cif file in CrystalMaker X, version 10.8.1). Thermal ellipsoids are drawn at a 50% occupancy level.

Slow evaporation of the solvent from a reaction mixture starting with excess Pd_3_(OAc)_6_ over **1b** gave crystals suitable for crystallographic analysis. The results indicate the presence of a cocrystal of both products **2b** and **8b** (Figure 6). This additional characterization provides additional confirmation of our structural assignments by NMR spectroscopic analysis (in solution). As far as we know, cocrystallization of this kind is unique in the chemistry of palladium(II) carboxylates, and only a few clusters^15^ or polymers^16^ containing more than four palladium atoms and a carboxylate group are known. On the other hand, there are approximately 20 examples of trinuclear Pd analogues^8,17^ of **8b**. Meanwhile, in **8b** and the aforementioned examples, three palladium atoms bridged by four acetates are arranged linearly with separations of 3 Å, where planes between the acetate ligands of neighboring palladium atoms are perpendicularly oriented. About a quarter of the reported structures exhibit other features, *e.g.*, three palladium atoms are bridged by less than four carboxylates and thus exhibit shorter separations or a bent structure.^18^ Four oxygen atoms around Pd4 in **8b** (Figure 6) are situated in a perfectly planar arrangement, completed by symmetry-related Pd3 atoms in a tetragonal bipyramid. In the case of the coordination polyhedra of Pd3 atoms, the situation is more complicated. Four heteroatoms of the ligands surrounding the Pd3 centers are partially distorted in the square plane of the metal, found 0.019(2)Å below the plane made by heteroatoms toward the Pd4 direction. The C23–Pd3–Pd4 angle is 117.40(12)°, a significant deviation from 90°. While there are typical weak noncovalent interactions, there is no significant connectivity between trinuclear and dinuclear molecules. There is a plethora of dinuclear palladium carboxylates from which about 20 structures contain a derivative of 2-[(*N*,*N*-dimethylaminoethyl)phenyl]-type ligands. There appears to be no major difference in these structures characterized by the square planar arrangement of the heteroatoms around palladium and relatively short interactions between metals of ∼3 Å.^4g,10,19^ The planes of the carboxylates are nearly perpendicular, with ligands occupying the remaining two coordination sites with an alternate arrangement. Ligand substitution has a negligible influence on the intramolecular N–Pd interaction, being ∼2 Å. The presence of fluorine atoms, especially in the *ortho*- and *para*-positions (6 and 4), in relation to palladium causes a bigger deflection of phenyl planes away from the ideal parallel orientation. For example, the interplanar angle in the unsubstituted ligand is 12.35(11)°,^10^ whereas the presence of fluorine in position 5 of **2b** promotes a change to 33.18(19)°. Further difluorination^10^ in positions 4 and 5 leads to an angle of 42.13(12)°. Alteration of the fluorine atoms, to either positions 3 and 6 or 4 and 6, widens the angles to 89.43(19) and 87.85(11)°, respectively.

**Figure 7:**
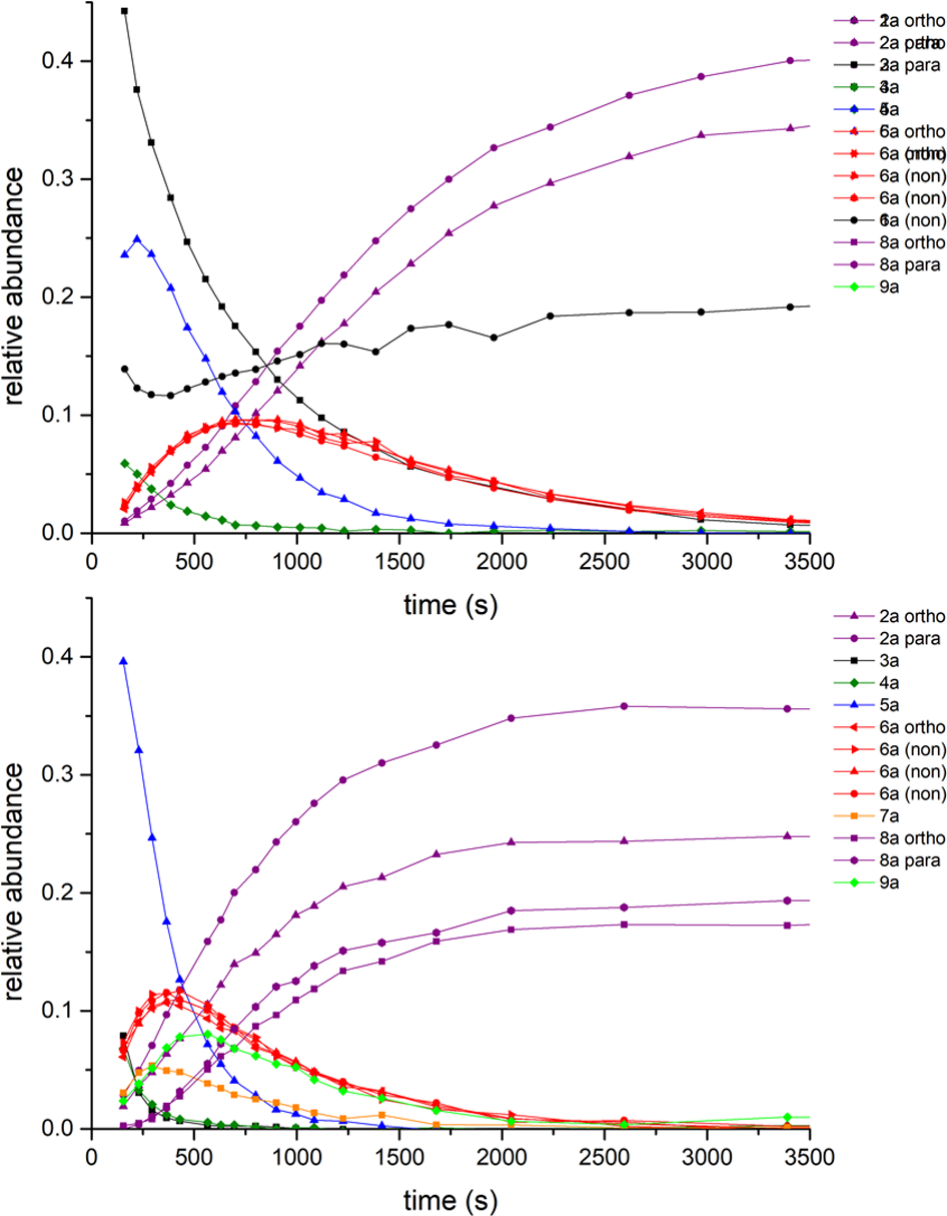
Comparison of the evolution profiles obtained from ^19^F{^1^H} NMR for reaction of different amounts (1.31 top and 0.5 bottom equiv) of **1a** with “Pd(OAc)_2_” (1 equiv) in CD_2_Cl_2_ at room temperature. The signals of products **2a**, **8a**, and **9a** are given for clarity.

With the knowledge gained about the reaction intermediates involving substrate **1b**, we measured the kinetic behavior of the 3-substituted substrate **1a** (a nonsymmetrical substrate) in CH_2_Cl_2_ and CH_3_CN. The experiment shows analogous behavior and intermediates, as in the case of symmetrical substrate **1b** (see Figure 7 and S22 – Supporting Information).

## Discussion and Conclusions

**Scheme 2:**
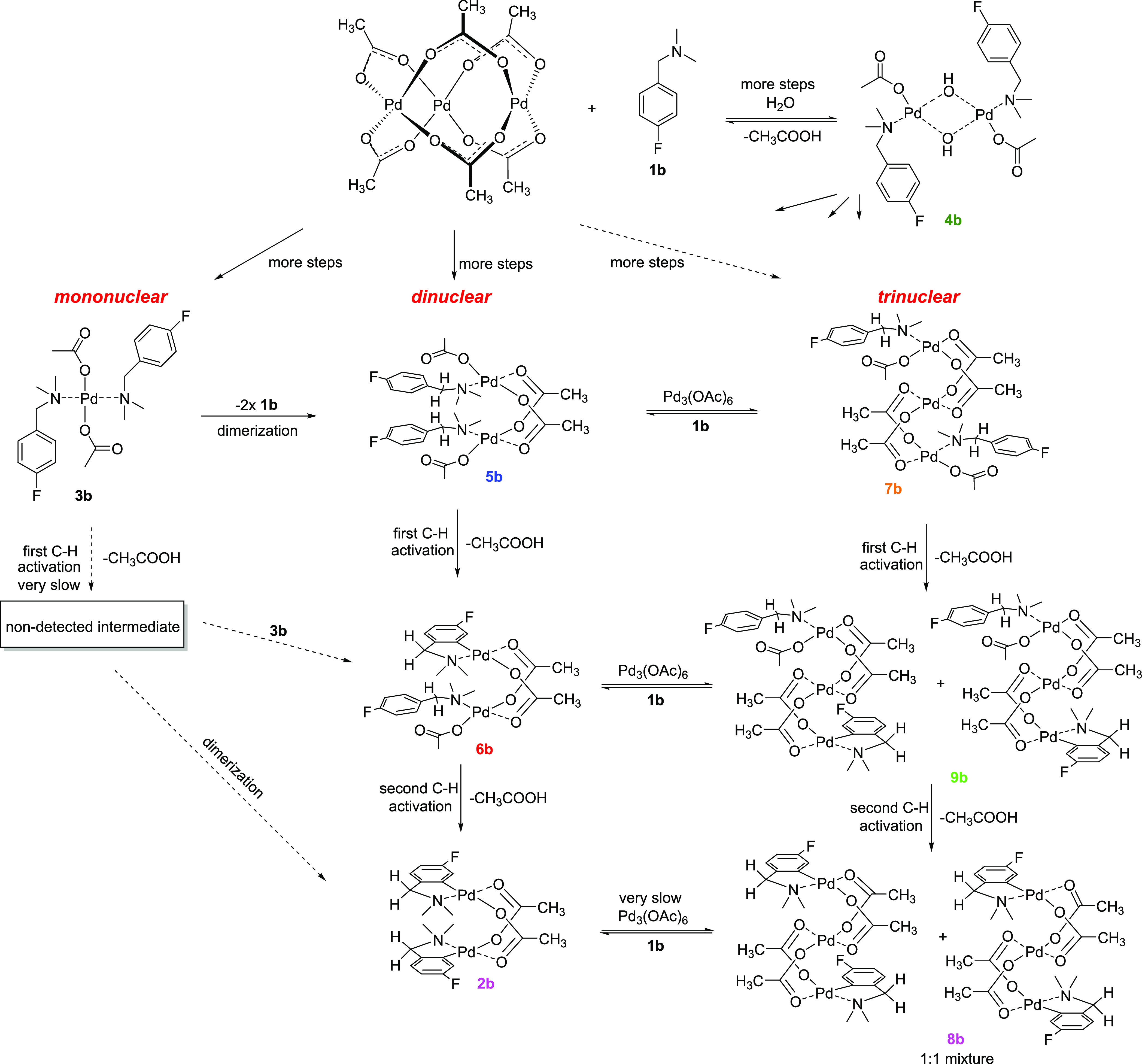
General Reaction Scheme Showing Mononuclear, Dinuclear, and Trinuclear Pathways for Reaction of Pd_3_(OAc)_6_ with **1b** We indicate the starting point as Pd_3_(OAc)_6_, which can be in equilibrium with dimer Pd_2_(OAc)_4_ and monomer Pd(OAc)_2_ species. We suspect that there are exchange processes involving water (and/or acetic acid).

We have shown that Pd_3_(OAc)_6_, which is a Pd cluster species, reacts with fluorinated substrates **1**, for which there is a dependence on the stoichiometry of the species **3**, **5**, and **7** formed (Scheme 2). It is likely that **3** is formed first, which is then subsequently transformed into **5** or **7** while releasing one molecule of the unreacted substrate **1** (Figure 5, top left). However, alternative direct formation of **5** or **7** is possible, especially in the presence of higher quantities of palladium. In the presence of H_2_O (*e.g.*, in the presence of adventitious moisture), **4** is formed. In specific terms relating to the fluorinated substrate **1b**, on formation of **5b**, it undergoes C–H bond activation to form **6b** followed by a subsequent second C–H bond activation to afford **2b**. To the best of our knowledge, this is the first time that a half-activated intermediate **6b** has been evidenced experimentally. Furthermore, it has been determined that **9** derives from **7**.

From the kinetic behavior (Figure 5), it was deduced that **7b** reaches a maximum later than analogue **5b**, and **9b** later than analogue **6b**. Furthermore, product **8b** appears following the formation of **2b**. We can state that the trinuclear Pd_3_(OAc)_6_ cluster interacts first with substrates **1**, which are subsequently broken down, fragmenting into mono- or dinuclear Pd species, from which the dinuclear Pd pathway can proceed (Scheme 2). However, in the presence of excess Pd, the dinuclear species reacts with Pd_3_(OAc)_6_, allowing the trinuclear intermediate **7b** to be formed. Species **7b** further undergoes two subsequent C–H bond activations within the trinuclear Pd framework via **9b** in forming **8b**.

Overall, there are three reaction pathways (mono-, di-, and trinuclear) whose significance is changing with the **1**/Pd_3_(OAc)_6_ ratio. The trinuclear Pd pathway becomes pronounced when using higher amounts of Pd_3_(OAc)_6_. Furthermore, the horizontal transformations (*e.g.*, between **6** and **9**), shown in Scheme 2, occur.

There are several factors speaking against the most common hypothesis that C–H activation occurs exclusively via mononuclear Pd_1_ species, while the polynuclear species are formed later. Firstly, the changes in the ratio of *ortho* and *para* activated products discussed above (see Figure 3 and Table 1) provide evidence that at least two of these pathways occur, while each of them takes place with a different preference for formation of *ortho* and *para* products. In agreement with experiments employing different solvents, the presence of large Pd clusters disfavors the *ortho*-palladation. Secondly, our data shows that the mononuclear pathway is slow (Figure S21, see the Supporting Information). If the reaction proceeds with more than one equivalent of **1b**, the reaction rate significantly decreases. Thirdly, we have determined that **2b** can be converted to **8b** by addition of Pd_3_(OAc)_6_, over several hours (note: we see no change after five minutes, see Figure S20, Supporting Information). However, this is slower in comparison to the reactions studied. Thus, this shows that intermediate **9b** is kinetically relevant.

In conclusion, we have provided experimental evidence that cyclopalladation reactions can proceed via dinuclear and trinuclear Pd cluster/complexes, in addition to mononuclear Pd species. Our reaction outcomes suggest that cyclopalladation is slower for mononuclear Pd species, involving substrates such as **1** in a reaction with Pd_3_(OAc)_6_. Regioselectivities are subtly different, depending on the reaction pathway. These findings highlight that there is an added complexity in cyclopalladation reactions of palladium(II) acetate with appropriate C–H substrates containing Pd-directing groups, a far-reaching finding with implications for catalysis and synthetic chemistry involving palladacyclic intermediates.

## Experimental Details

### Influence of the Solvent

To the solution of 10 mg (0.045 mmol) of “Pd(OAc)_2_” in 0.5 mL of the solvent was added 8 μL (0.059 mmol) of **1a**, and the reaction mixture was stirred at room temperature. After 5 h, the solvent was evaporated, and the residue was dissolved in CDCl_3_ and analyzed by ^1^H and ^19^F proton-decoupled NMR. Next, 4 drops of pyridine was added into the NMR tube, and the sample was analyzed again.

### Influence of Reaction Stoichiometry

To 10 mg (0.045 mmol) of “Pd(OAc)_2_” was added 0.5 mL of the solvent (chloroform stabilized with EtOH 1%, acetonitrile, and DCM) followed by various amounts (2, 4, 6, 8, and 12 μL) of **1a**. The reaction mixture was stirred for 4 h at room temperature, the solvent was evaporated, and the residue was dissolved in CDCl_3_ and analyzed by ^1^H and ^19^F proton-decoupled NMR. Next, 4 drops of pyridine was added into the NMR tube, and the sample was analyzed again.

### Kinetic Experiments

Palladium acetate (10 mg, 0.045 mmol) was dissolved in an NMR tube in 0.55 mL of a deuterated solvent (CD_3_CN or CD_2_Cl_2_). After measurement of the first spectra, 8, 5, 3, or 2^20^ μL of **1b** was added, and the ^1^H and ^19^F kinetics was followed. For the 2D experiments, the reaction mixture was cooled to −20 °C 7 min after initiation.

### Crystal Growth

The cocrystal of **2b** and **8b** was prepared by the slow evaporation of a solution containing 20 mg (0.09 mmol) of “Pd(OAc)_2_” and 5 μL of **1b** in 0.5 mL of DCM in a pentane atmosphere. The single-crystal X-ray diffraction data can be found deposited to the Cambridge Crystallographic Database Centre (CCDC 2189187).

## Supporting Information Available

Further experimental details and spectral data (PDF)
