# Supplementary material for: Direct Cyclopalladation of Fluorinated Benzyl Amines by Pd3(OAc)6: The Coexistence of Multinuclear Pdn Reaction Pathways Highlights the Importance of Pd Speciation in C–H Bond Activation
Source: Organometallics. 2023 May 31;42(16):2197–205. doi: 10.1021/acs.organomet.3c00178 (PMC10466454; doi:10.1021/acs.organomet.3c00178)
Supplement: Supplementary file 1 — om3c00178_si_001.pdf [file om3c00178_si_001.pdf]

Supporting Information

Direct Cyclopalladation of Fluorinated Benzyl Amines by $Pd_3(OAc)_6$: The Co-Existence of Multi-Nuclear Pd_n Reaction Pathways Highlights the Importance of Pd Speciation in C-H Bond Activation

Ian J. S. Fairlamb,[†] Jan Lang,[#] Aleš Růžička,[§] Miloš Sedlák,[†] Jiří Váňa,*[‡]*

* Joint corresponding authors. ian.fairlamb@york.ac.uk; jiri.vana@upce.cz

[†]Department of Chemistry, University of York, Heslington, York YO10 5DD, U.K.

[#]Department of Low Temperature Physics, Faculty of Mathematics and Physics, Charles University, V Holešovičkách 747/2, 18000 Prague 8, Czech Republic

[§]Department of General and Inorganic Chemistry, Faculty of Chemical Technology, University of Pardubice, Studentská573, 53210 Pardubice, Czech Republic

[‡]Institute of Organic Chemistry and Technology, Faculty of Chemical Technology, University of Pardubice, Studentská573, 53210 Pardubice, Czech Republic

Contents

General Experimental Details 2
Influence of the solvent 2
Temperature effect 6
Influence of reaction stoichiometry 7
Kinetic experiments 8
Water effect 19
Reversibility test 20
Characterization of the compounds 23
Crystallography 24
Representative Spectral Data 26
References 30

General Experimental Details

All of chemicals and solvents were purchased from Acros Organics, Sigma-Aldrich, or Fluorochem and used as received. Palladium(II) diacetate was purchased from Sigma-Aldrich – recrystalized. FT-IR spectra were recorded on FT-IR Nicolet iS50 using the ATR technique (diamond). NMR spectra were measured at room temperature on Bruker AVANCE III 400 or Bruker Ascend™ 500. ^{1}H NMR spectra were calibrated to tetramethylsilane or toluene (impurity in palladium(II) diacetate which proved useful for the kinetic experiments). ^{13}C NMR spectra were calibrated to the middle signal of the multiplet for the solvent used. All NMR spectra were processed using Bruker TopSpin software.

Influence of the solvent

To a solution containing 10 mg (0.045 mmol) of 'Pd(OAc)$_2$' in 0.5 ml of solvent was added 8 μL (0.059 mmol) of **1a**. The reaction mixture was stirred at room temperature. After 5 hours the solvent was removed *in vacuo*, and the residue dissolved in $CDCl_3$ and analyzed by ^{1}H and ^{19}F proton decoupled NMR spectroscopic analysis. Next, 4 drops of pyridine was added into the NMR tube and the sample was analyzed again (addition of pyridine allows the mononuclear palladium(II) palladacyclic adducts to be characterized). The integral intensities of signals corresponding to each of the regioisomeric products, obtained after addition of pyridine, are shown Table S1, overleaf.

Table S1 Influence of solvent on the ratio of regioisomers $\mathbf{2_{a6}}$ and $\mathbf{2_{a2}}$.[1]

	$^{19}F\{^{1}H\}$ integral intensities		
**Solvent**	$\mathbf{2a_6}$ (*para*)	$\mathbf{2a_2}$ (*ortho*)	Unreacted **1a**
1,4-dioxane (ε = 2.21)	1	0.50	0.16
Benzene (ε = 2.27)	1	0.55	0.16
$CHCl_3$/1% EtOH (ε = 4.89)	1	0.74	0.13
$CDCl_3$/Ag foil (ε = 4.89)	1	0.73	0.13
Chlorobenzene (ε = 5.62)	1	0.73	0.09
AcOH (ε = 6.17)	1	0.67	3.58[a]
THF (ε = 7.58)	1	0.73	0.20
DCM (ε = 8.93)	1	0.86	0.19
HFIP (ε = 16.7)	1	0.83	0.19
Acetone (ε = 20.56)	1	0.88	0.16
EtOH (ε = 24.55)	1	0.85[b]	0.71
CH_3OH (ε = 32.66)	1	0.79	0.14
Nitromethane (ε = 35.87)	1	0.87	0.21
Acetonitrile (ε = 35.94)	1	0.95	0.25
Dimethylsulfoxide (ε = 46.45)	1	1.17	0.43[c]

[a] very slow reaction and low conversion.
[b] poor solubility of '$Pd(OAc)_2$' in solvent, black precipitate, unreacted substrate clearly visible.
[c] after 12 hours at 50°C, broad signals, could be different complex.

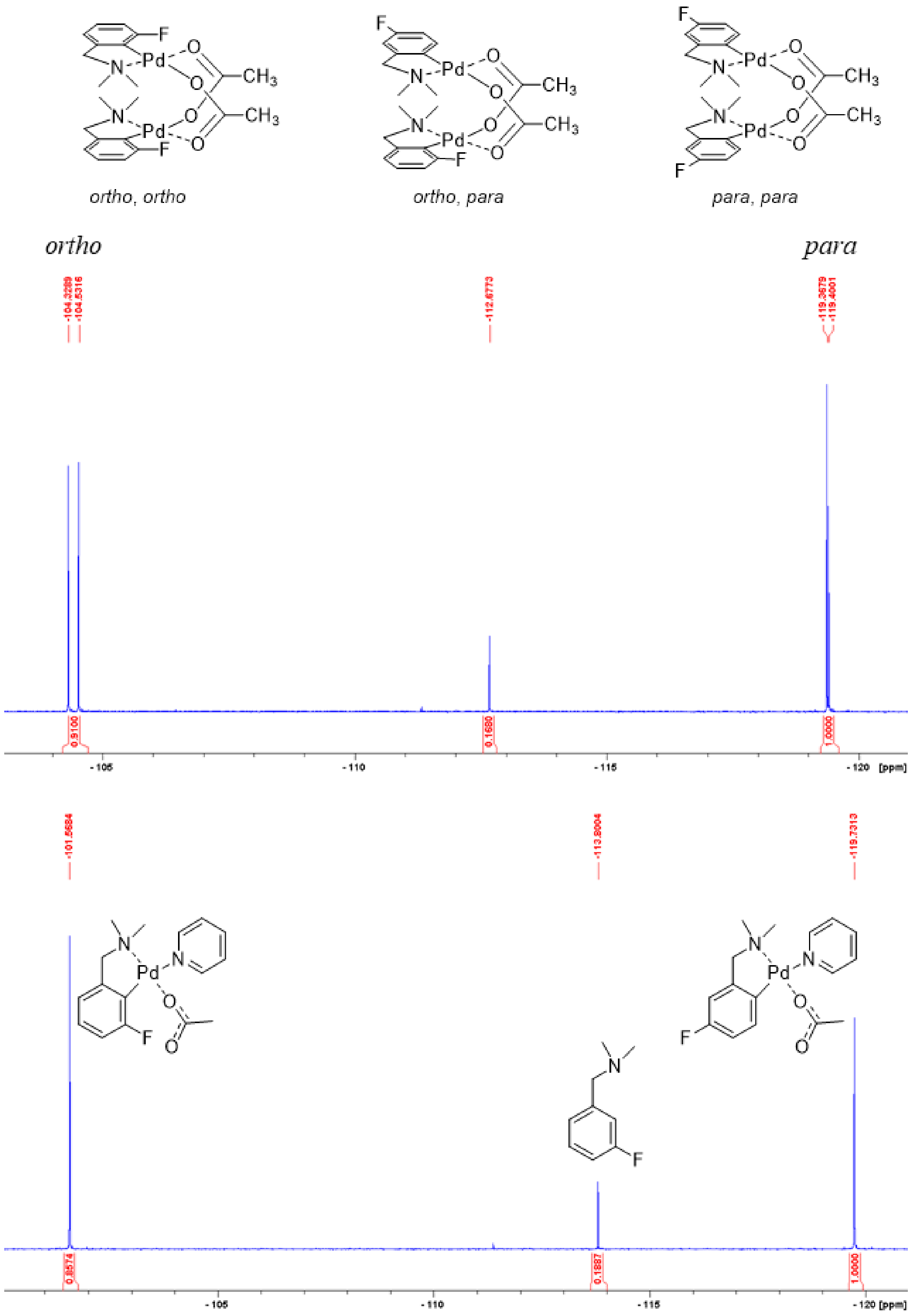

Figure S1. Typical $^{19}F\{^{1}H\}$ NMR spectra used for determination of solvent influence. Top: spectrum of isomers of **2a** prepared in DCM, measured in $CDCl_3$. Bottom: spectrum after addition of pyridine.

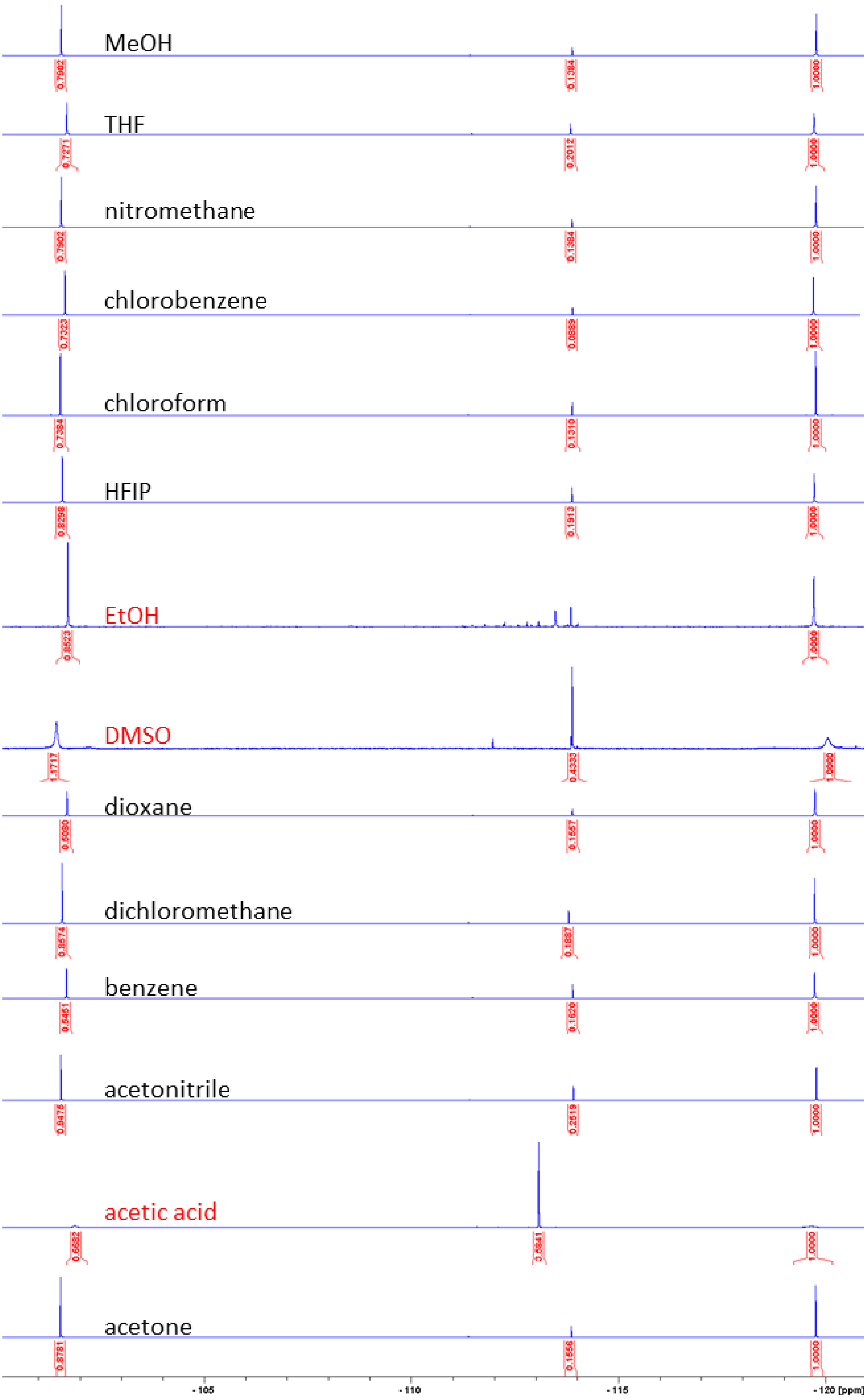

Figure S2. Comparison of $^{19}F\{^1H\}$ NMR spectra after addition of pyridine used for determination of solvent influence.

Temperature effect

To a solution of 10 mg (0.045 mmol) of ‘$Pd(OAc)_2$’ in 0.5 ml of solvent, tempered to the desired temperature, was added 8 µL (0.059 mmol) of **1a**. The reaction mixture was stirred for 5 hours. Next, the solvent was removed *in vacuo*, and the residue was dissolved in $CDCl_3$ and analyzed by ^1H and ^{19}F proton decoupled NMR spectroscopic analysis. Next, 4 drops of pyridine was added into the NMR tube and the sample was analyzed again. The integral intensities of signals corresponding to each of the regioisomeric products, obtained after addition of pyridine, are shown Table S2, below.

Table S2 Temperature effect on the ratio of regioisomers $\mathbf{2_{a6}}$ and $\mathbf{2_{a2}}$.

**acetonitrile**					
	$^{19}F\{^1H\}$ integral intensities			^1H integral intensities	
Temperature (°C)	$\mathbf{2a_6}$ (*para*)	$\mathbf{2a_2}$ (*ortho*)		$\mathbf{2a_6}$ (*para*)	$\mathbf{2a_2}$ (*ortho*)
22	1	0.96		1	0.95
40	1	0.96		1	0.98
60	1	0.96		1	0.96
80	1	0.94		1	0.96
**toluene**					
Temperature (°C)	$\mathbf{2a_6}$ (*para*)	$\mathbf{2a_2}$ (*ortho*)		$\mathbf{2a_6}$ (*para*)	$\mathbf{2a_2}$ (*ortho*)
22	1	0.51		1	0.5
40	1	0.54		1	0.52
60	1	0.54		1	0.53
80	1	0.56		1	0.53
100	1	0.53		1	0.53
110	1	0.53		1	0.51

Influence of reaction stoichiometry

To 10 mg (0.045 mmol) of ‘$Pd(OAc)_2$’ was added 0.5 ml of solvent (chloroform-stabilized with EtOH 1%, acetonitrile, DCM), followed by varying amounts of **1a** (2, 4, 6, 8, 12, 16 μL). The reaction mixture was stirred 4 hours at room temperature, and then the solvent was removed *in vacuo.* The resultant residue was dissolved in $CDCl_3$ and analyzed by ^1H and ^{19}F proton decoupled NMR spectroscopic analysis. Next, 4 drops of pyridine was added into the NMR tube and the sample was analyzed again, as described earlier.

Table S3 Stoichiometric effect on the ratio of regioisomers $\mathbf{2_{a6}}$ and $\mathbf{2_{a2}}$.

**acetonitrile**					
	$^{19}F\{^1H\}$ integral intensities			^1H integral intensities	
molar ratio **1a**:‘$Pd(OAc)_2$’	$\mathbf{2a_6}$ (*para*)	$\mathbf{2a_2}$ (*ortho*)		$\mathbf{2a_6}$ (*para*)	$\mathbf{2a_2}$ (*ortho*)
0.33:1	1	0.85		1	0.83
0.66:1	1	0.87		1	0.88
0.99:1	1	0.92		1	0.91
1.31:1	1	0.95		1	0.94
1.99:1	1	0.95		1	0.97
2.60:1	1	0.99		1	0.96
**dichloromethane**					
molar ratio **1a**:‘$Pd(OAc)_2$’	$\mathbf{2a_6}$ (*para*)	$\mathbf{2a_2}$ (*ortho*)		$\mathbf{2a_6}$ (*para*)	$\mathbf{2a_2}$ (*ortho*)
0.33:1	1	0.77		1	0.78
0.66:1	1	0.80		1	0.82
0.99:1	1	0.83		1	0.83
1.31:1	1	0.85		1	0.86
1.99:1	1	0.89		1	0.89
2.60:1	1	0.93		1	0.90
**chloroform**					
molar ratio **1a**:‘$Pd(OAc)_2$’	$\mathbf{2a_6}$ (*para*)	$\mathbf{2a_2}$ (*ortho*)		$\mathbf{2a_6}$ (*para*)	$\mathbf{2a_2}$ (*ortho*)
0.33:1	1	0.73		1	0.71
0.66:1	1	0.72		1	0.72
0.99:1	1	0.72		1	0.74
1.31:1	1	0.74		1	0.74
1.99:1	1	0.76		1	0.76

Kinetic experiments

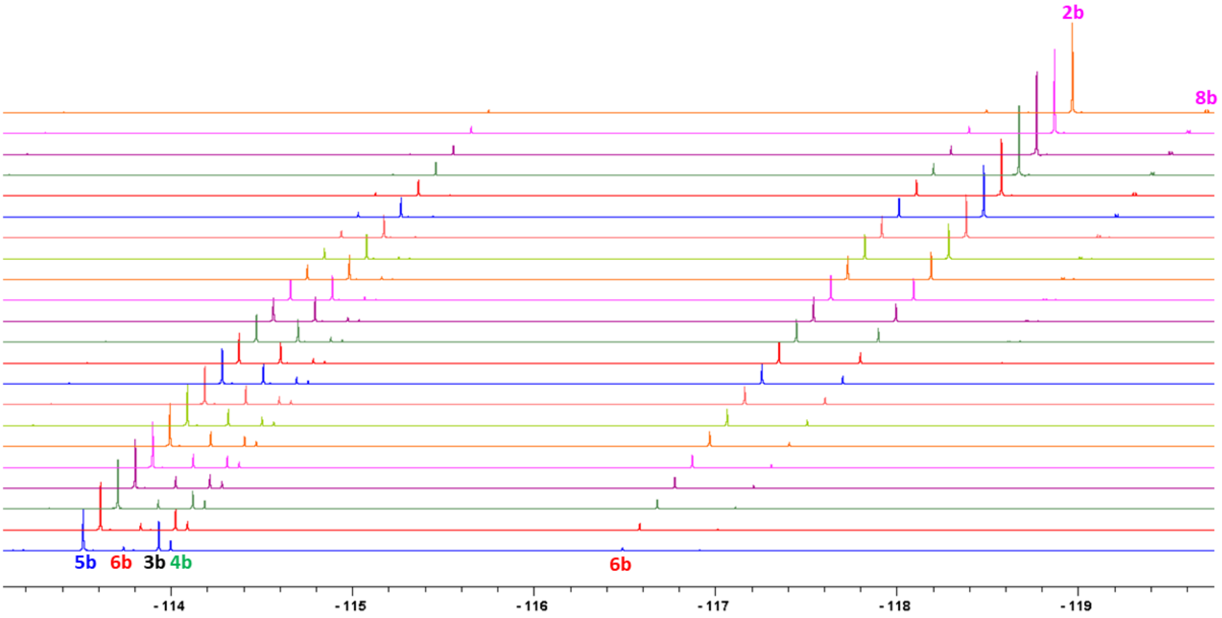

Figure S3. The time dependent $^{19}F\{^{1}H\}$ NMR spectra of reaction of 8 µL (1.29 eqv.) of **1b** and 10 mg of 'Pd(OAc)$_2$' in CD_2Cl_2 at room temperature.

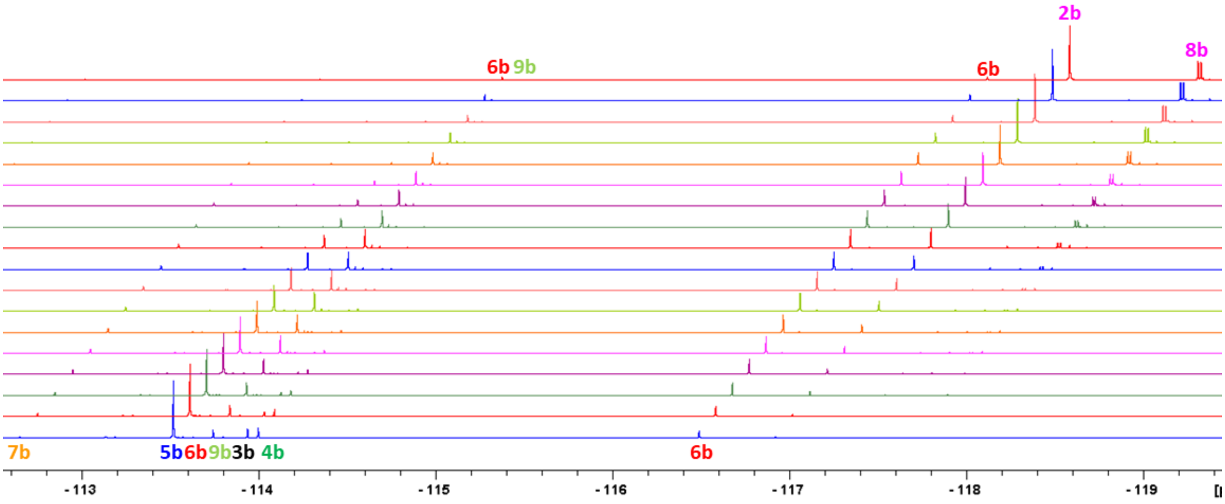

Figure S4. The time dependent $^{19}F\{^{1}H\}$ NMR spectra of reaction of 5 µL (0.85 eqv.) of **1b** and 10 mg of 'Pd(OAc)$_2$' in CD_2Cl_2 at room temperature.

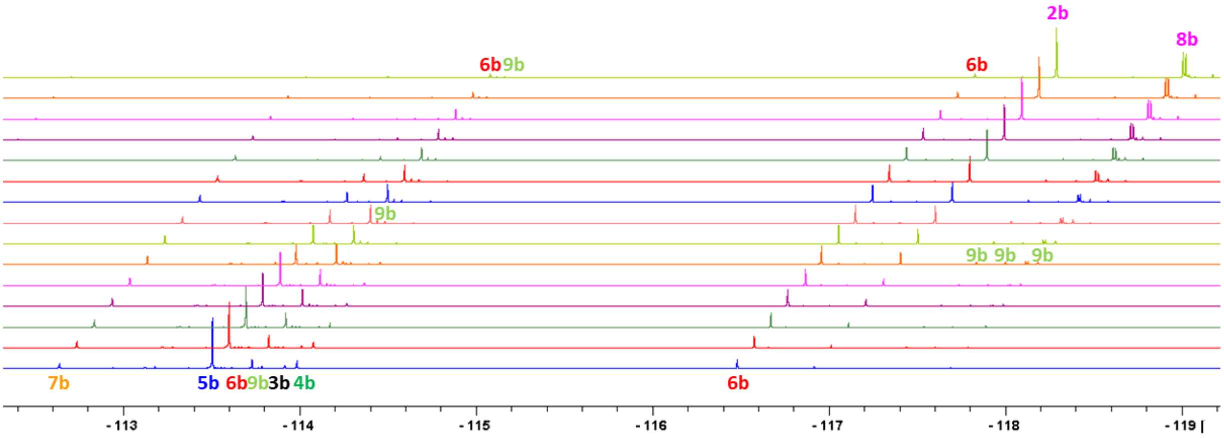

Figure S5. The time dependent $^{19}F\{^{1}H\}$ NMR spectra of reaction of 3 μL (0.53 eqv.) of **1b** and 10 mg of 'Pd(OAc)$_2$' in CD_2Cl_2 at room temperature.

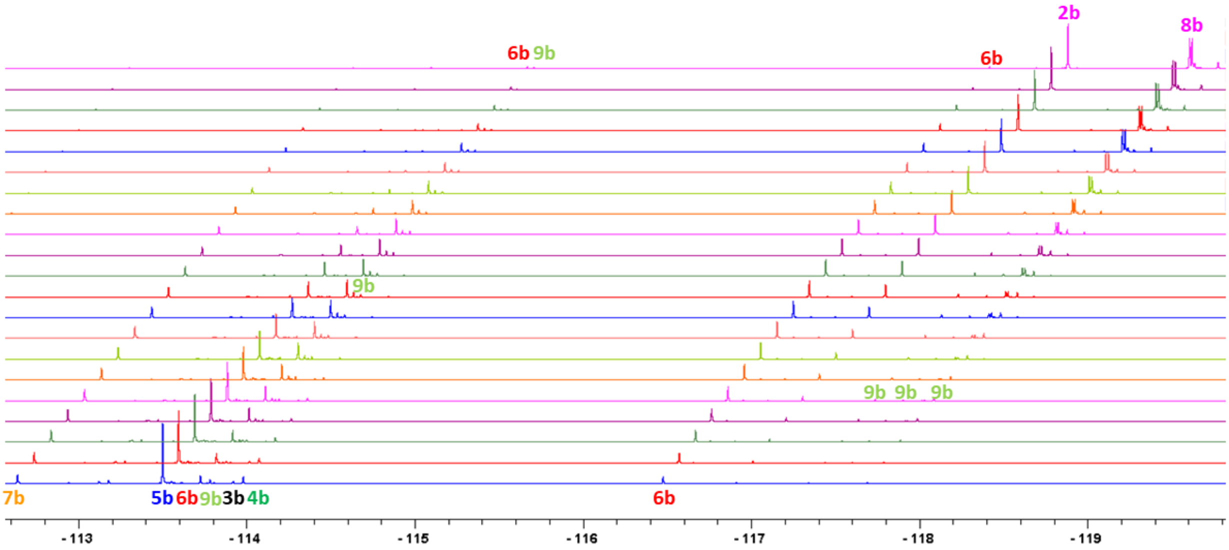

Figure S6. The time dependent $^{19}F\{^{1}H\}$ NMR spectra of reaction of 4 μL (0.34 eqv.) of **1b** and 20 mg of 'Pd(OAc)$_2$' in CD_2Cl_2 at room temperature.

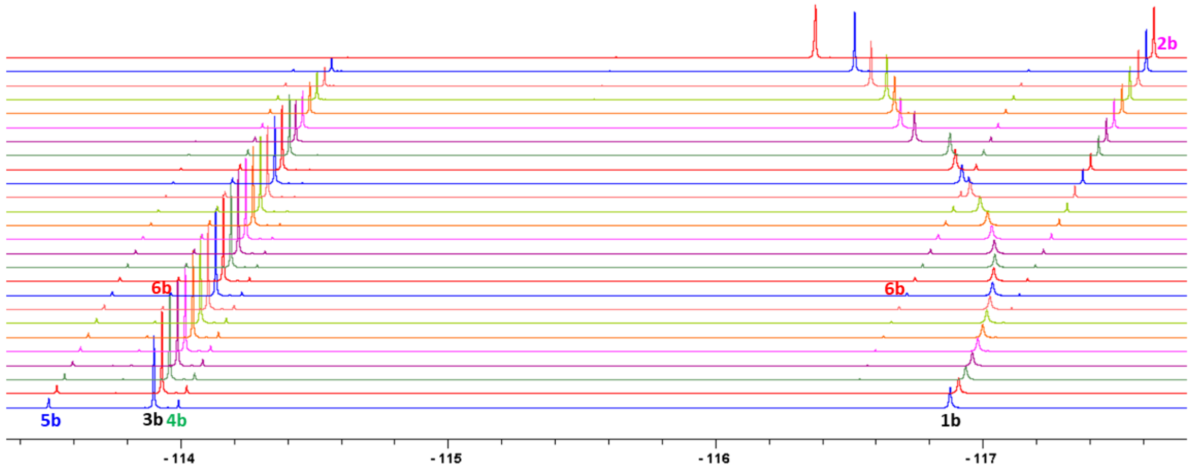

Figure S7. The time dependent $^{19}F\{^{1}H\}$ NMR spectra of reaction of 16 μL (2.6 eqv.) of **1b** and 10 mg of 'Pd(OAc)$_2$' in CD_2Cl_2 at room temperature.

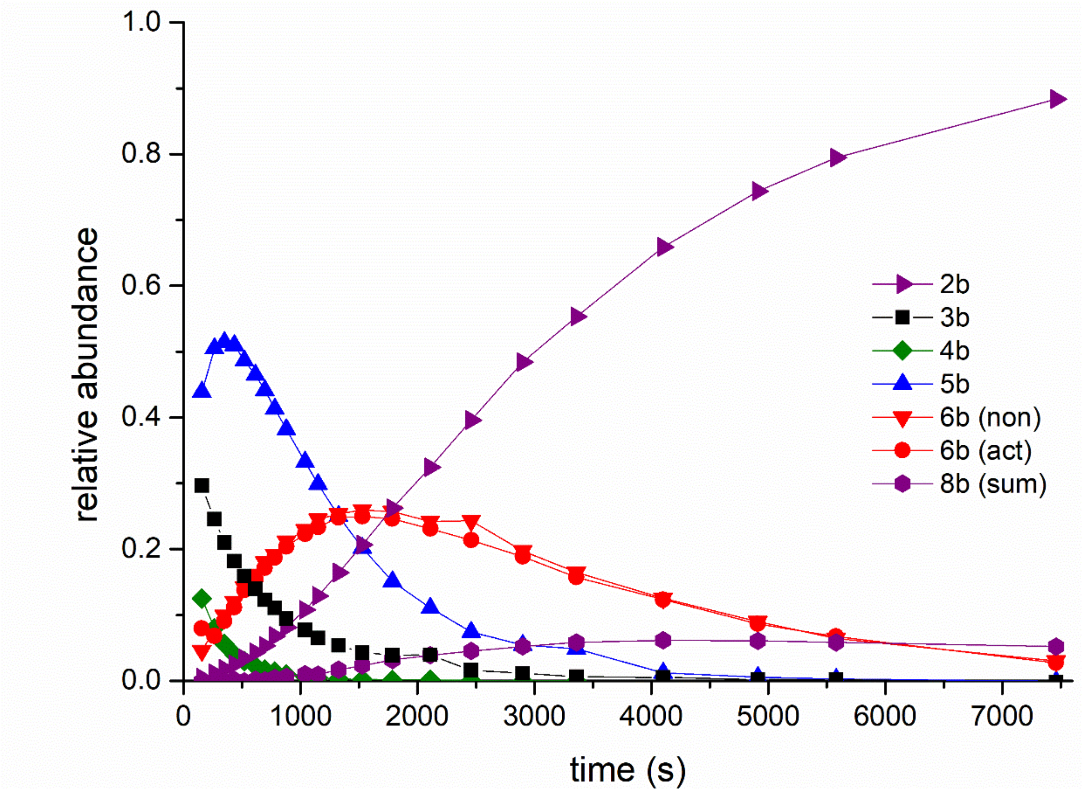

Figure S8. The signal evolution profile obtained from $^{19}F\{^{1}H\}$ NMR for reaction of 8 μL (1.29 eqv.) of **1b** and 10 mg of 'Pd(OAc)$_2$' in CD_2Cl_2 at room temperature.

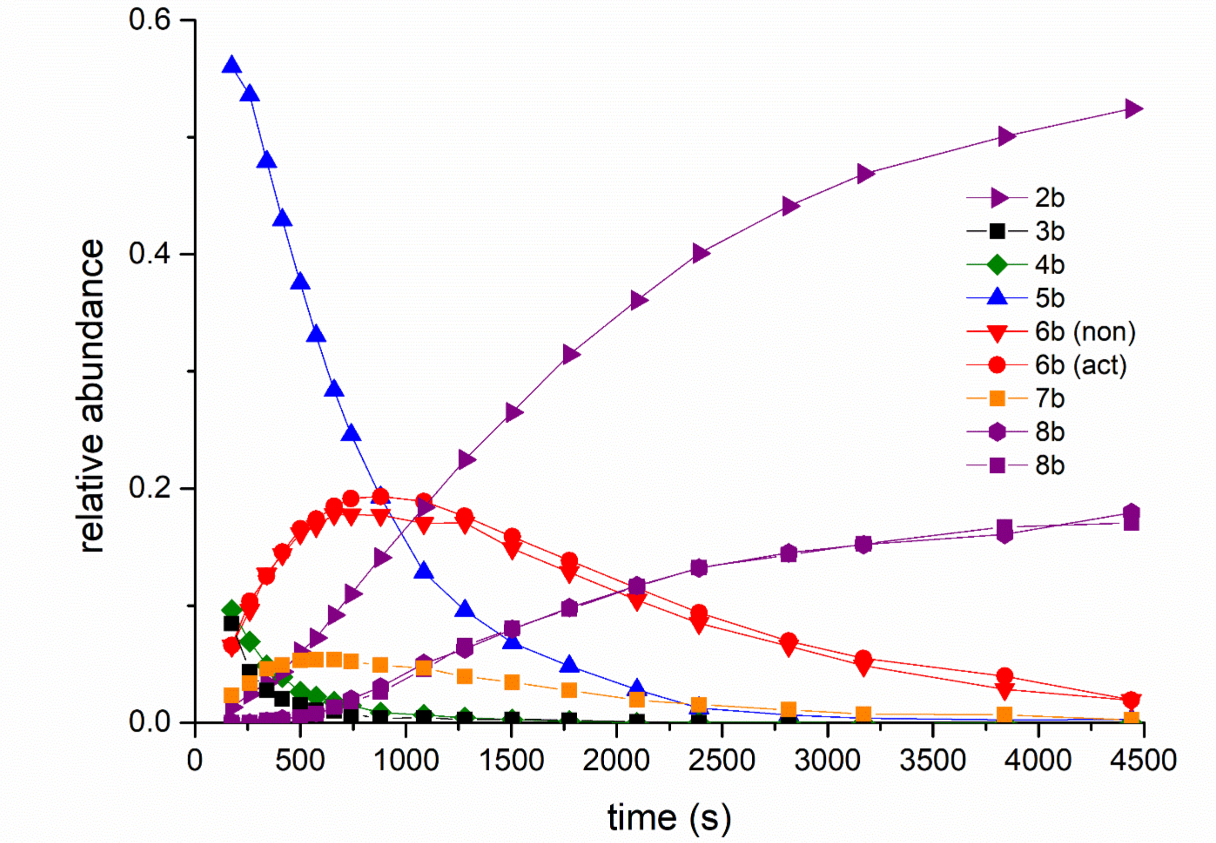

Figure S9. The signal evolution profile obtained from $^{19}F\{^{1}H\}$ NMR for reaction of 5 μL (0.85 eqv.) of **1b** and 10 mg of 'Pd(OAc)$_2$' in CD_2Cl_2 at room temperature.

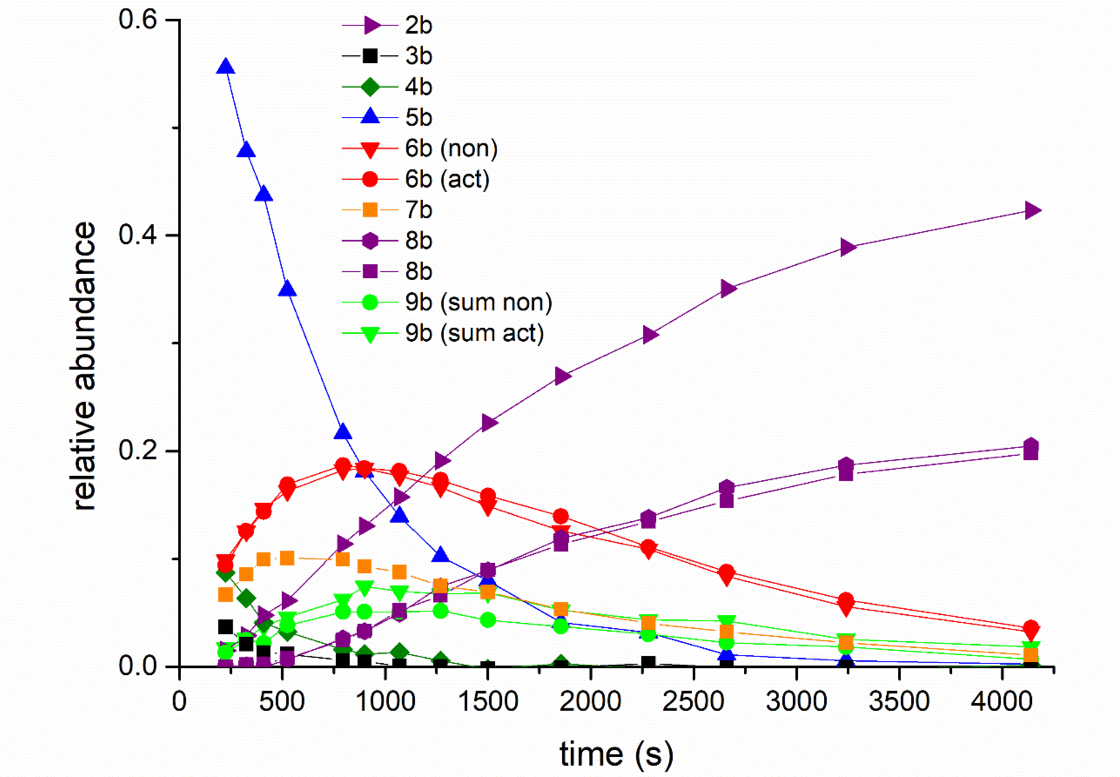

Figure S10. The signal evolution profile obtained from $^{19}F\{^{1}H\}$ NMR for reaction of 3 μL (0.53 eqv.) of **1b** and 10 mg of 'Pd(OAc)$_2$' in CD_2Cl_2 at room temperature.

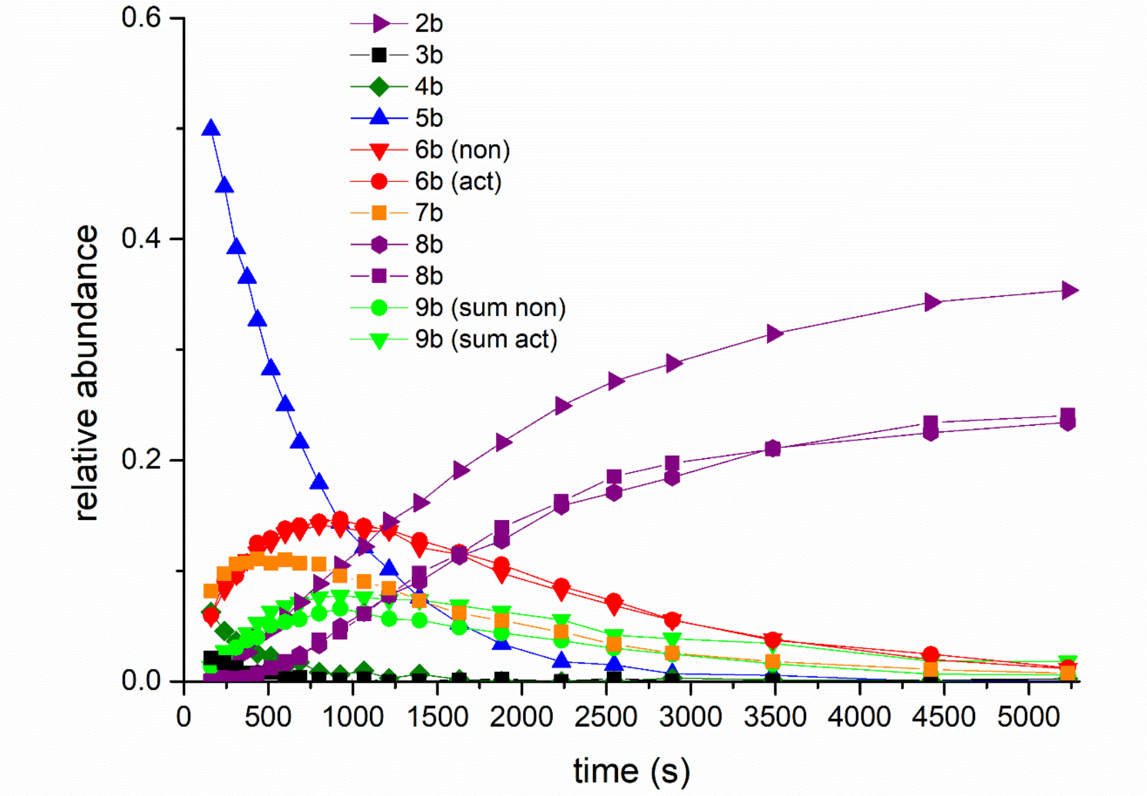

Figure S11. The signal evolution profile obtained from $^{19}F\{^{1}H\}$ NMR for reaction of 4 μL (0.34 eqv.) of **1b** and 20 mg of 'Pd(OAc)$_2$' in CD_2Cl_2 at room temperature.

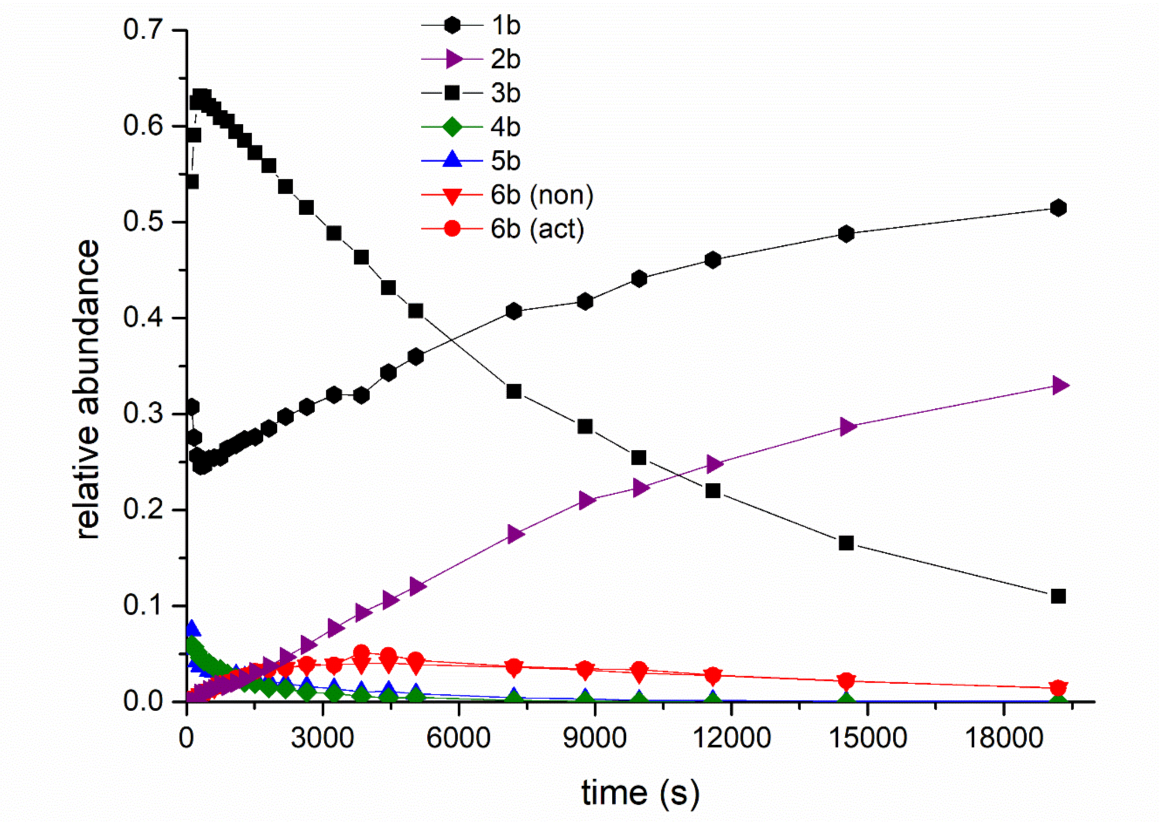

Figure S12. The signal evolution profile obtained from $^{19}F\{^{1}H\}$ NMR for reaction of 16 μL (2.6 eqv.) of **1b** and 10 mg of 'Pd(OAc)$_2$' in CD_2Cl_2 at room temperature.

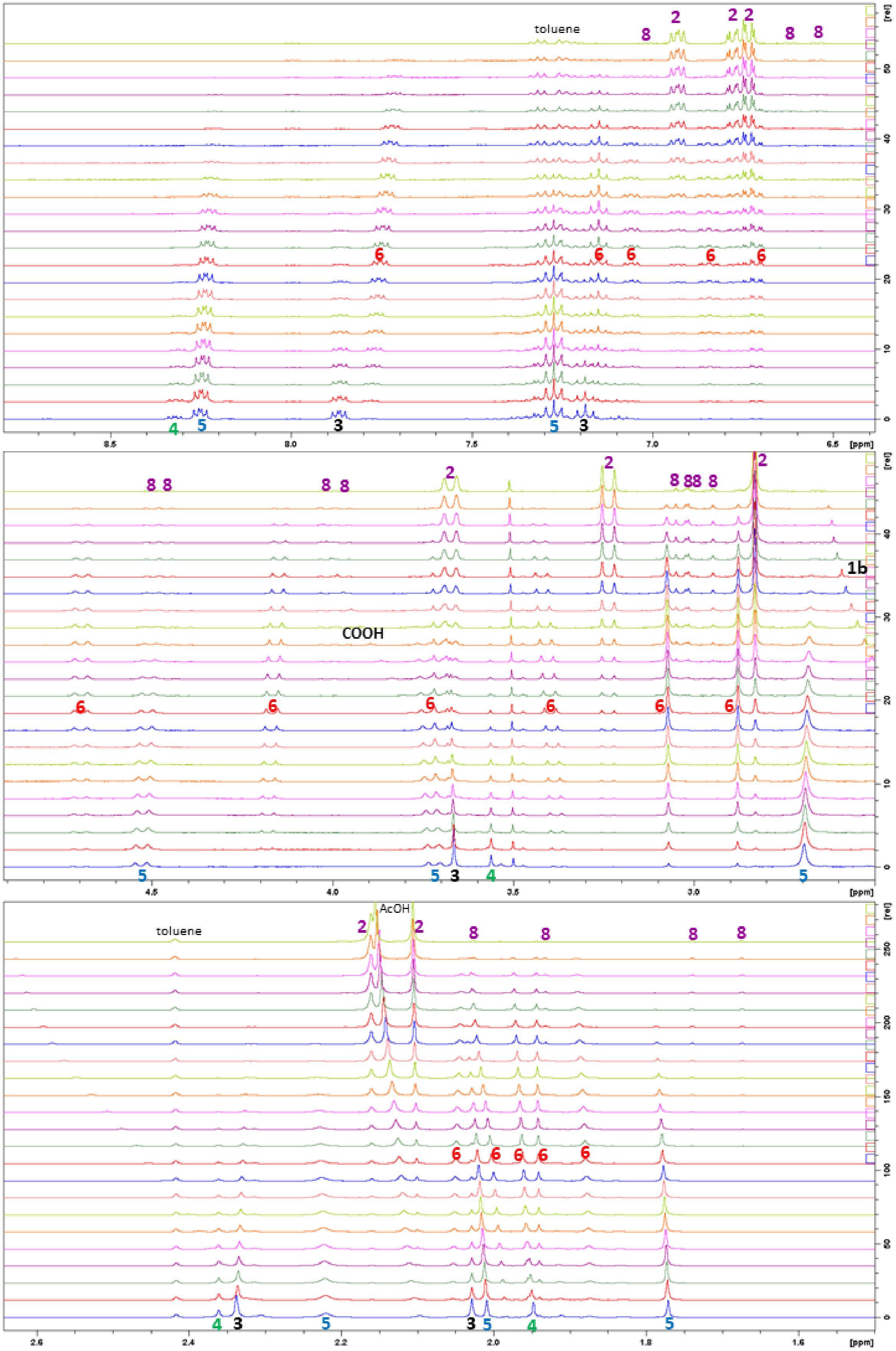

Figure S13. The ^{1}H NMR signal assignment for reaction of 8 μL (1.29 eqv.) of **1b** and 10 mg of 'Pd(OAc)$_2$' in CD_2Cl_2 at room temperature.

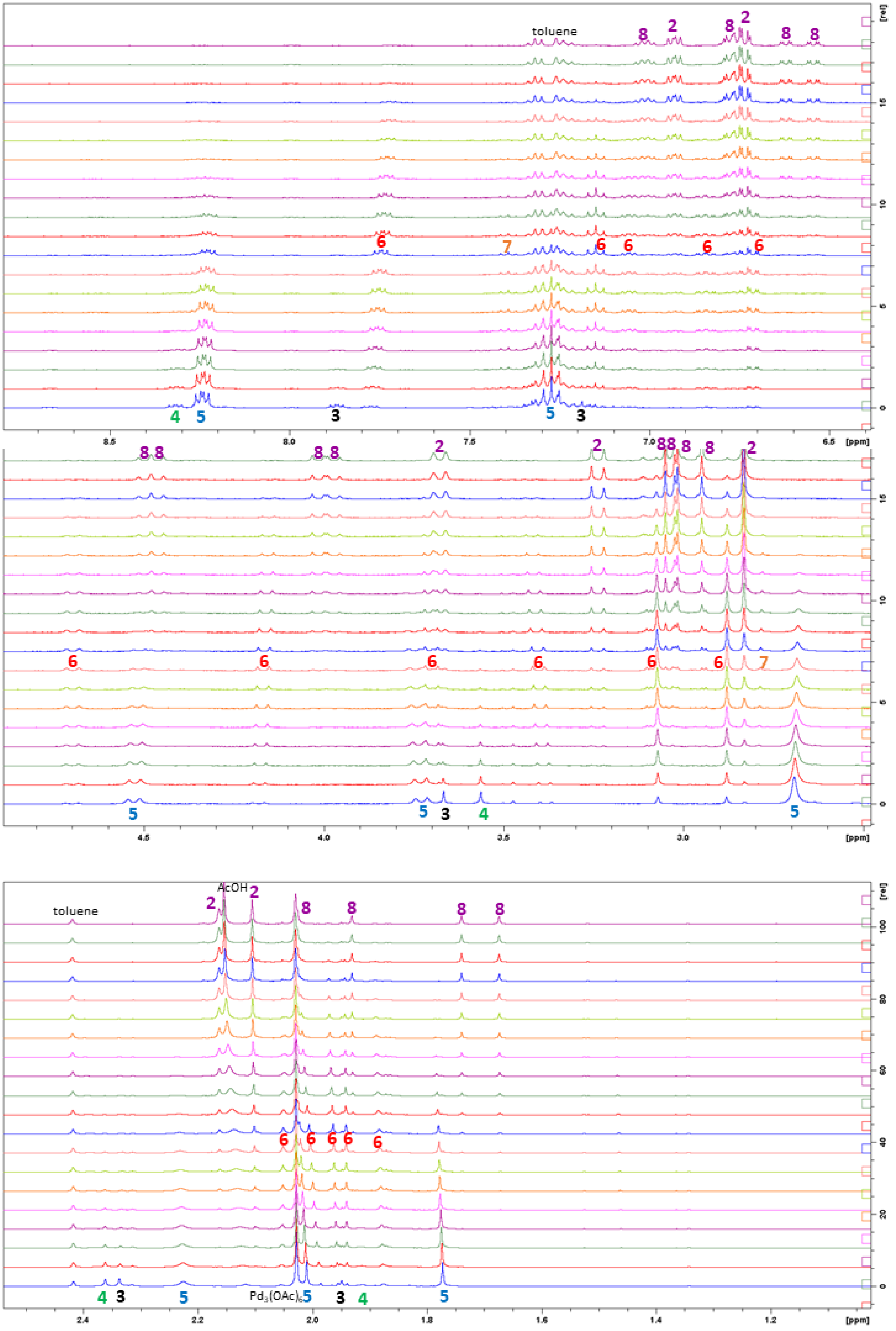

Figure S14. The ^{1}H NMR signal assignment for reaction of 5 µL (0.85 eqv.) of **1b** and 10 mg of ‘$Pd(OAc)_2$’ in CD_2Cl_2 at room temperature.

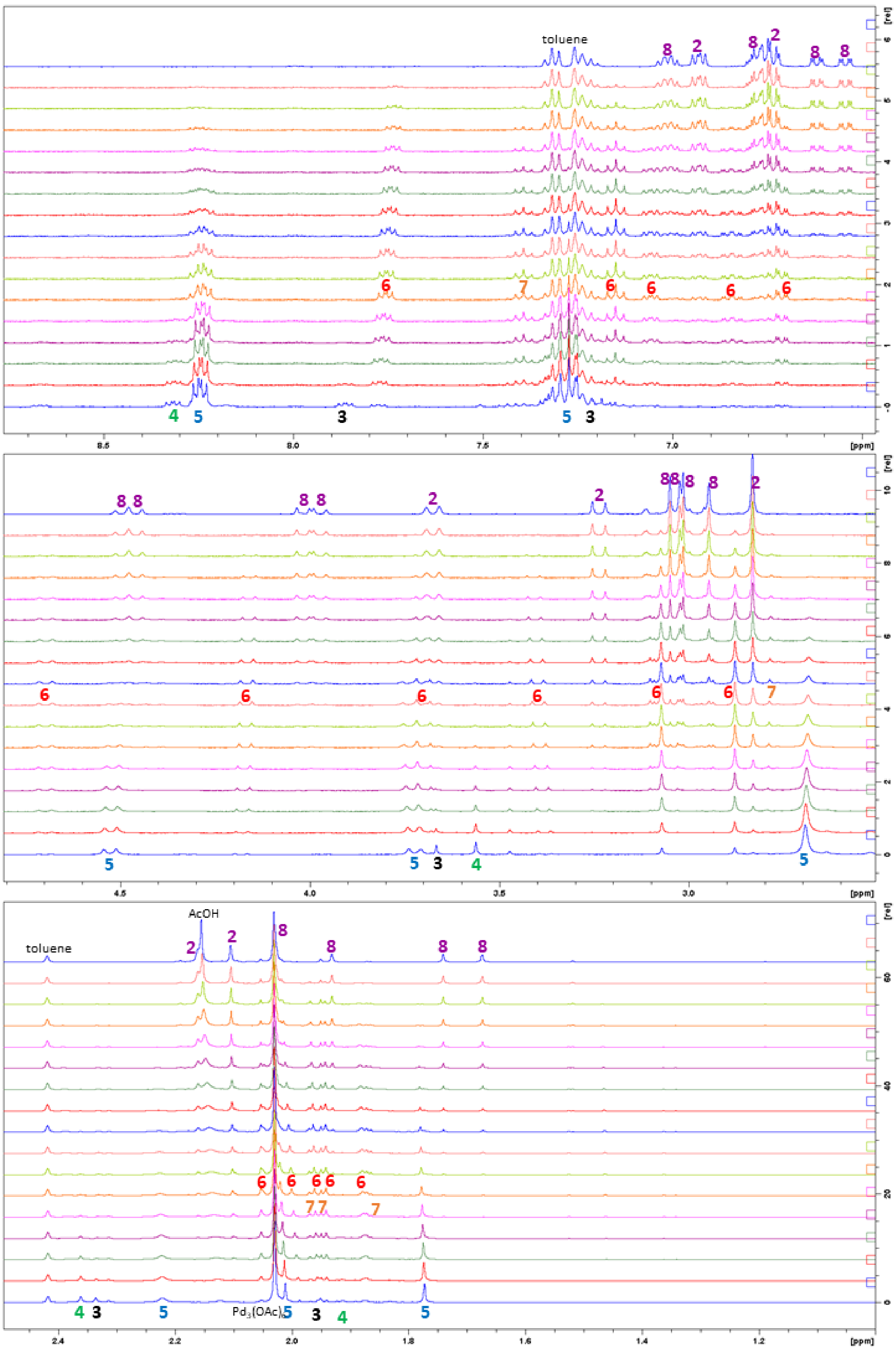

Figure S15. The ^{1}H NMR signal assignment for reaction of 3 μL (0.53 eqv.) of **1b** and 10 mg of ‘$Pd(OAc)_2$’ in CD_2Cl_2 at room temperature.

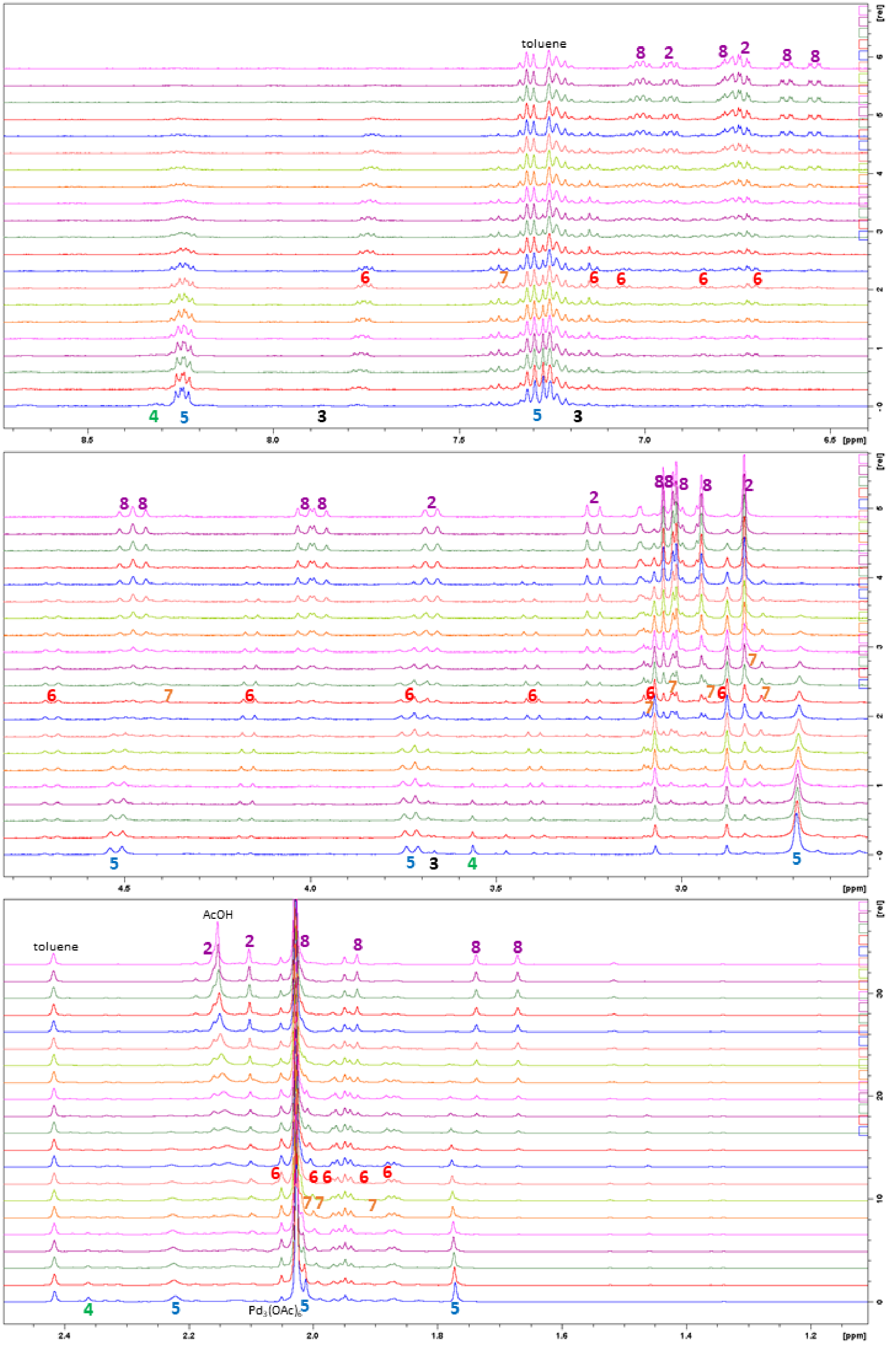

Figure S16. The ^{1}H NMR signal assignment for reaction of 4 μL (0.34 eqv.) of **1b** and 20 mg of 'Pd(OAc)$_2$' in CD_2Cl_2 at room temperature.

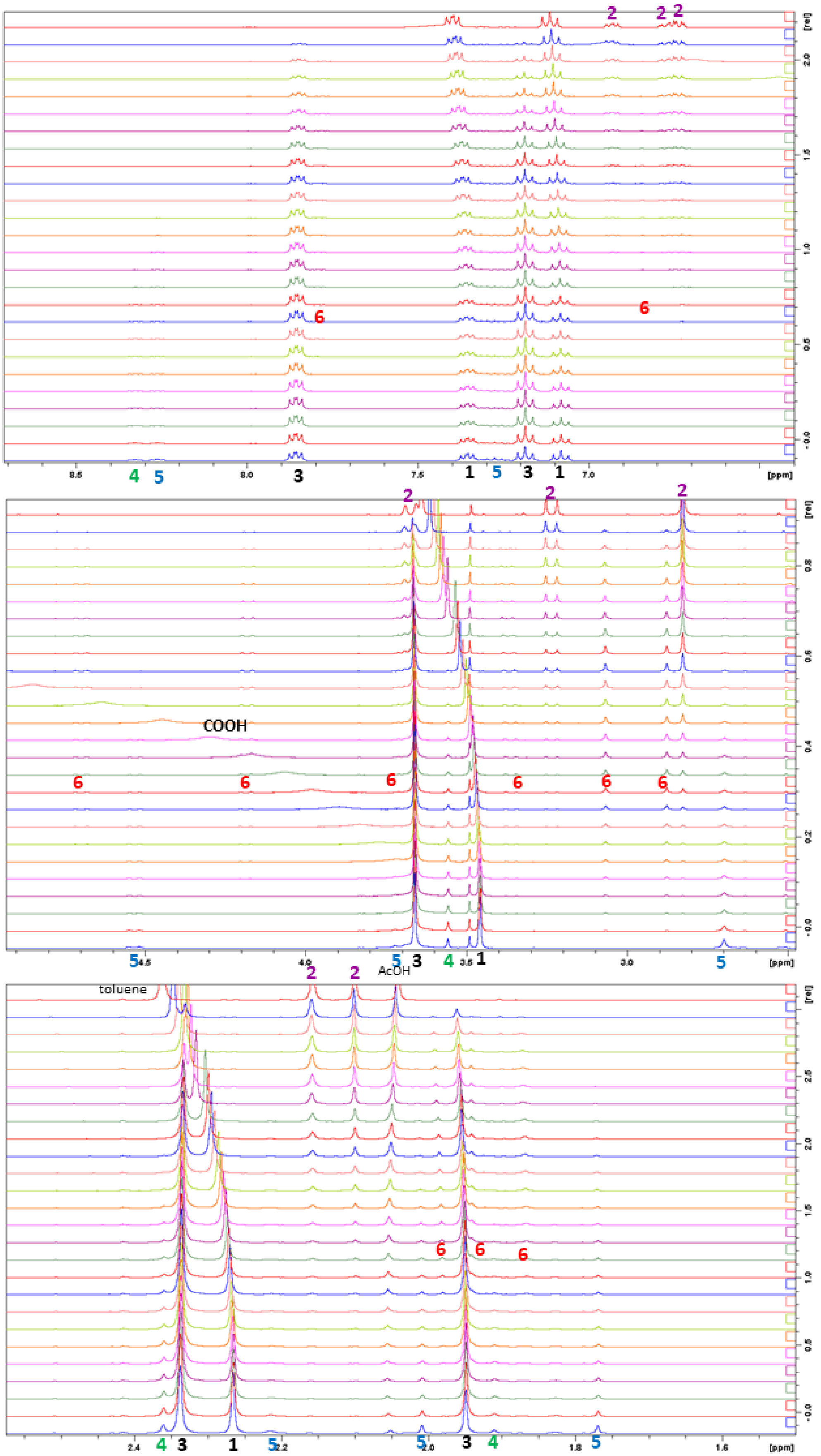

Figure S17. The ^{1}H NMR signal assignment for reaction of 16 μL (2.6 eqv.) of **1b** and 10 mg of 'Pd(OAc)$_2$' in CD_2Cl_2 at room temperature.

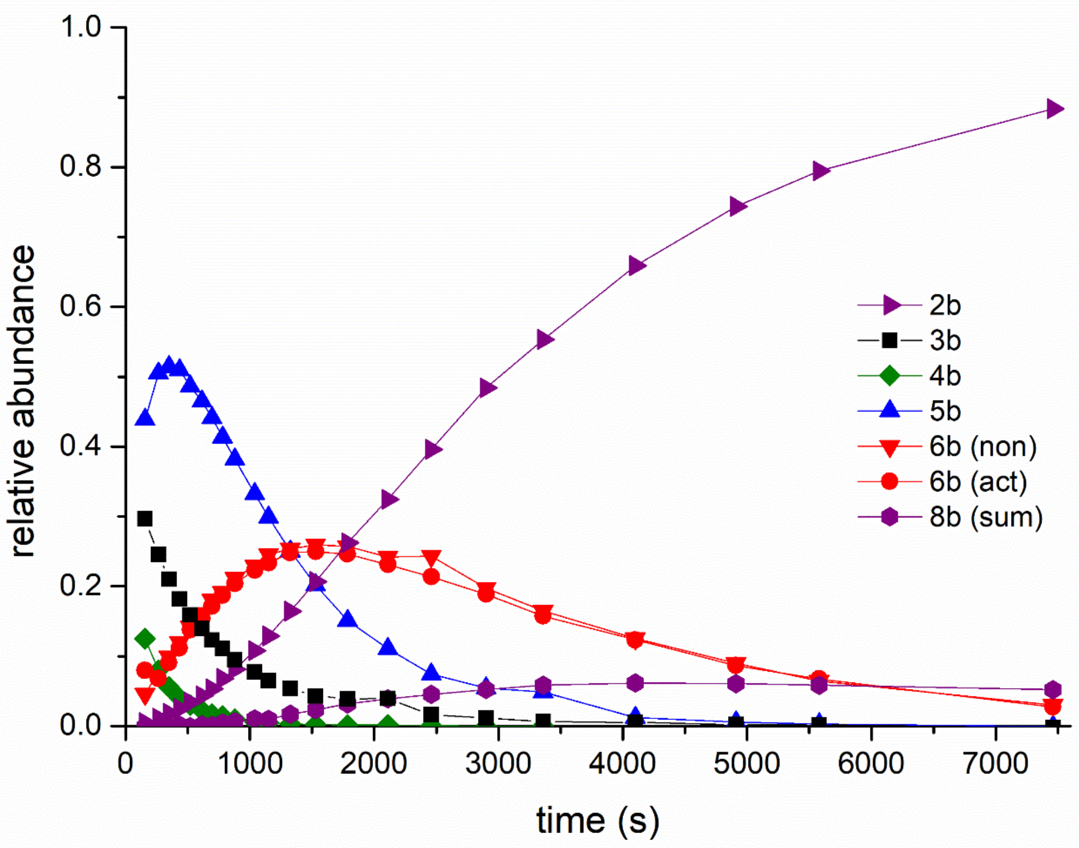

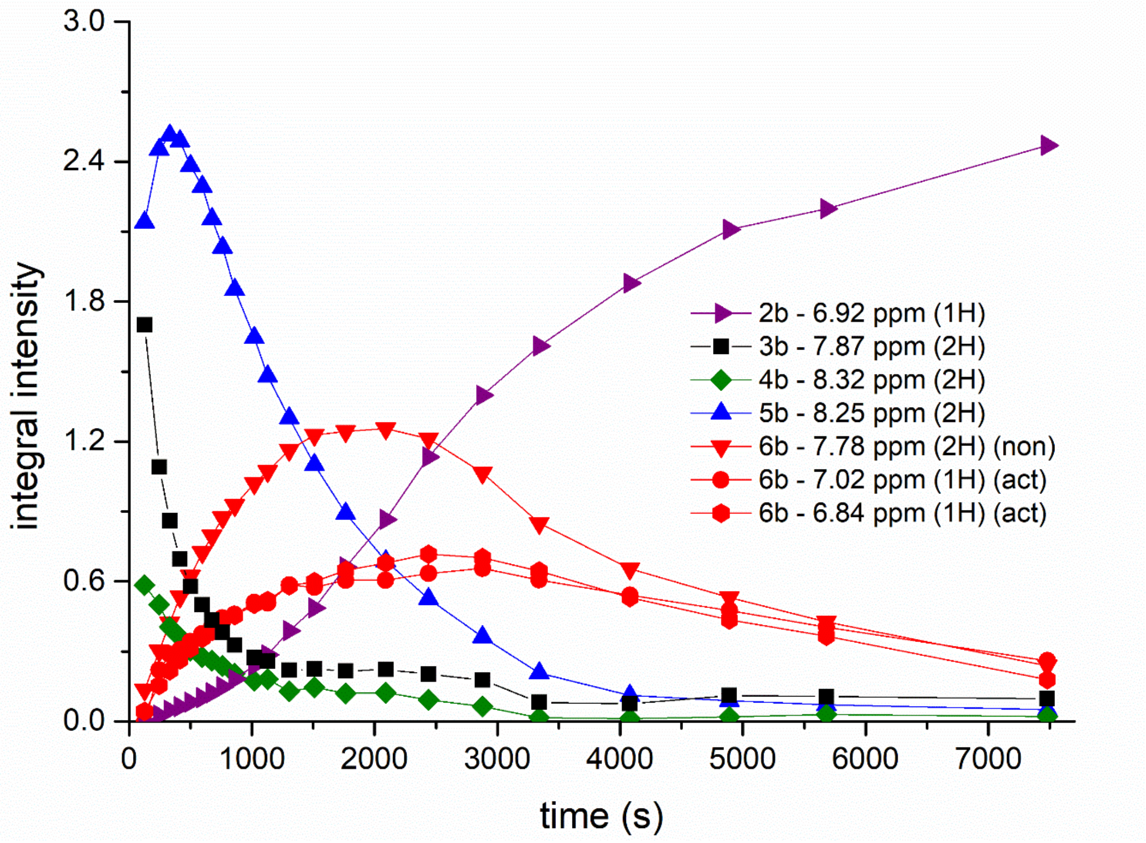

Figure S18. Comparison of the signal evolution profiles obtained from ^{1}H (aromatic region) (top) and ^{19}F{^{1}H} (bottom) NMR for reaction of 8 μL (1.29 eqv.) of **1b** and 10 mg of 'Pd(OAc)$_2$' in CD_2Cl_2 at room temperature.

Water effect

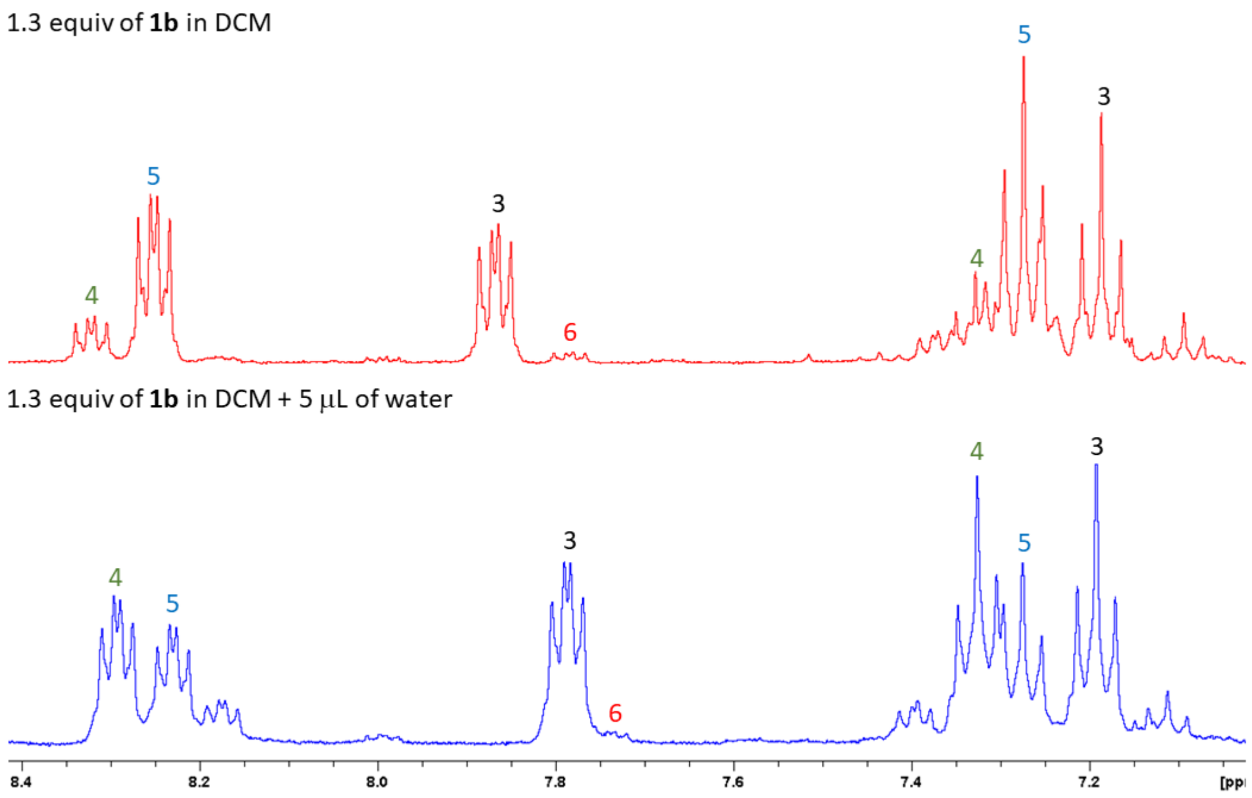

Figure S19. Comparison of the ^{1}H NMR spectra of the aromatic region for the reaction of 8 μL (1.29 eqv.) of **1b** and 10 mg of 'Pd(OAc)$_2$' in CD_2Cl_2 (top), and in CD_2Cl_2 with 5 μL of water (bottom) measured 2 minutes after initiation of the reaction.

Reversibility test

To a NMR tube containing a sample of the reaction of 10 mg (0.045 mmol) of ‘$Pd(OAc)_2$’ in 0.5 ml CD_2Cl_2 with 8 µL (1.3 equiv., 0.059 mmol) of **1b** was added 5 mg of ‘$Pd(OAc)_2$’. Spectral data was collected after 5 minutes and 48 hours.

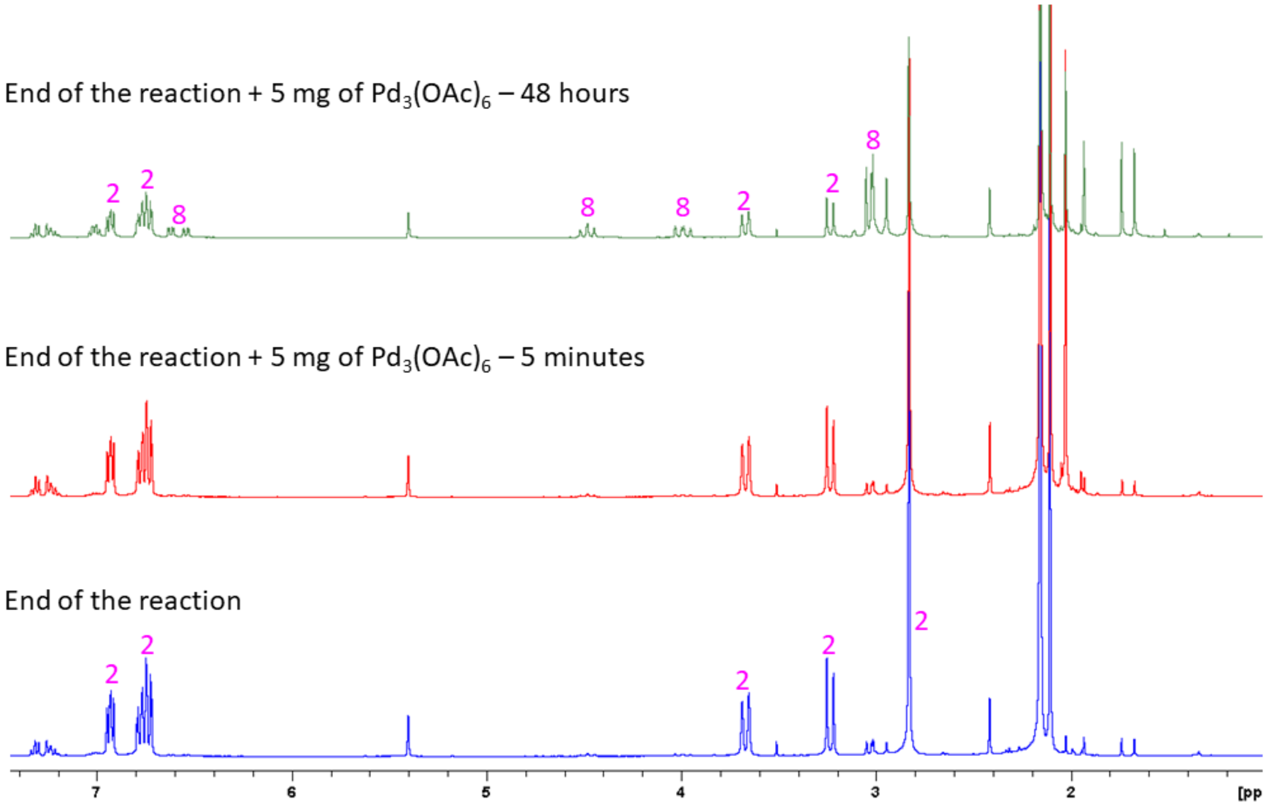

Figure S20. Comparison of the ^{1}H NMR for the end of the reaction of 8 µL (1.29 eqv.) of **1b** and 10 mg of ‘$Pd(OAc)_2$’ in CD_2Cl_2 (bottom) and 5 minutes (middle) and 48 hours (top) after addition of 5 mg ‘$Pd(OAc)_2$’.

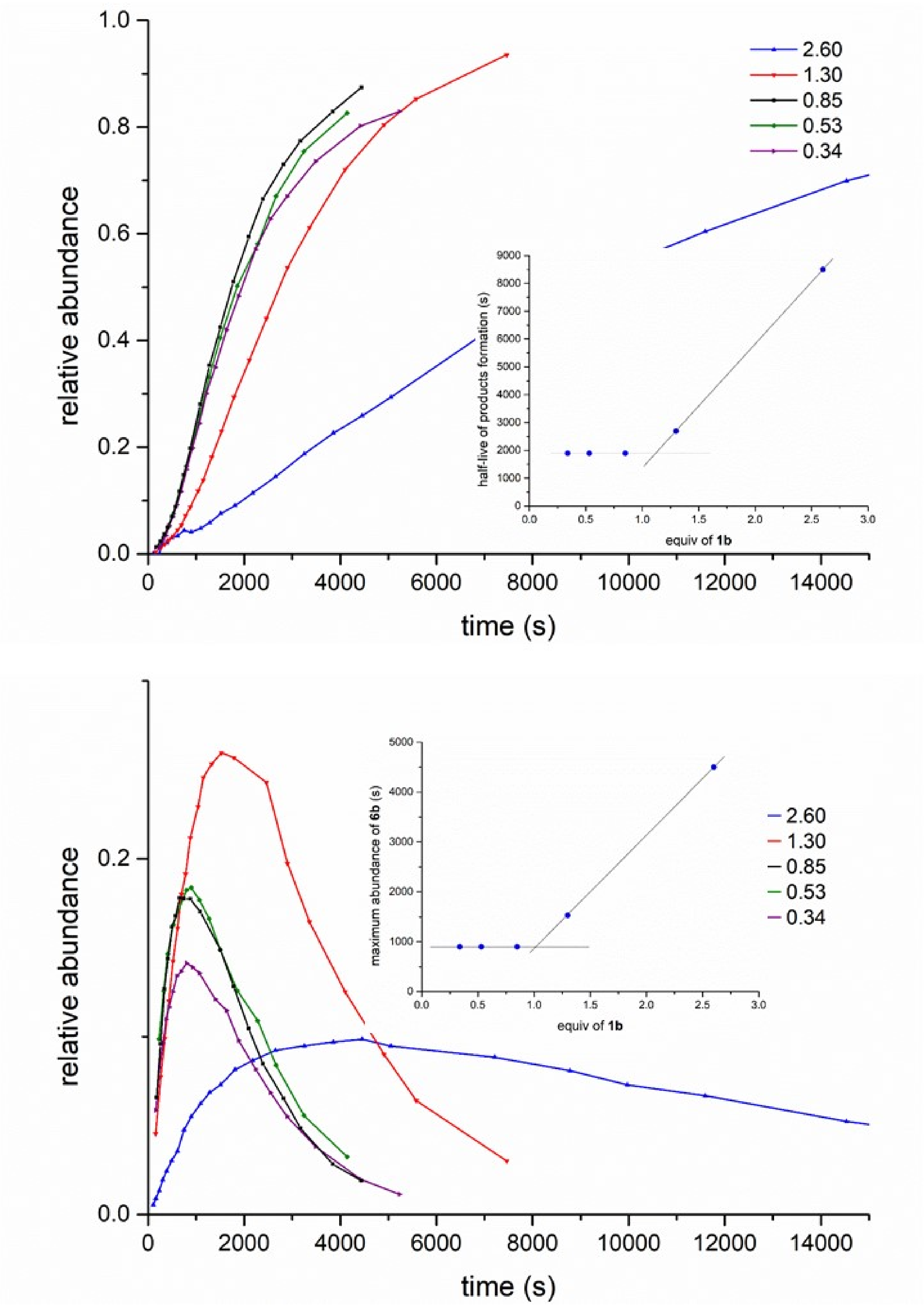

Figure S21. Comparison of the signal evolution profiles for sum of products (**2b**+**8b**) (top) and intermediate **6b** (bottom) obtained from experiments with different amounts of **1b** added to 10 mg of ‘Pd(OAc)$_2$’. The insets show dependences of ‘half-lives’ of products formation (top) or maximal abundances of **6b** (bottom) on the **1b**/‘Pd(OAc)$_2$’ ratio.

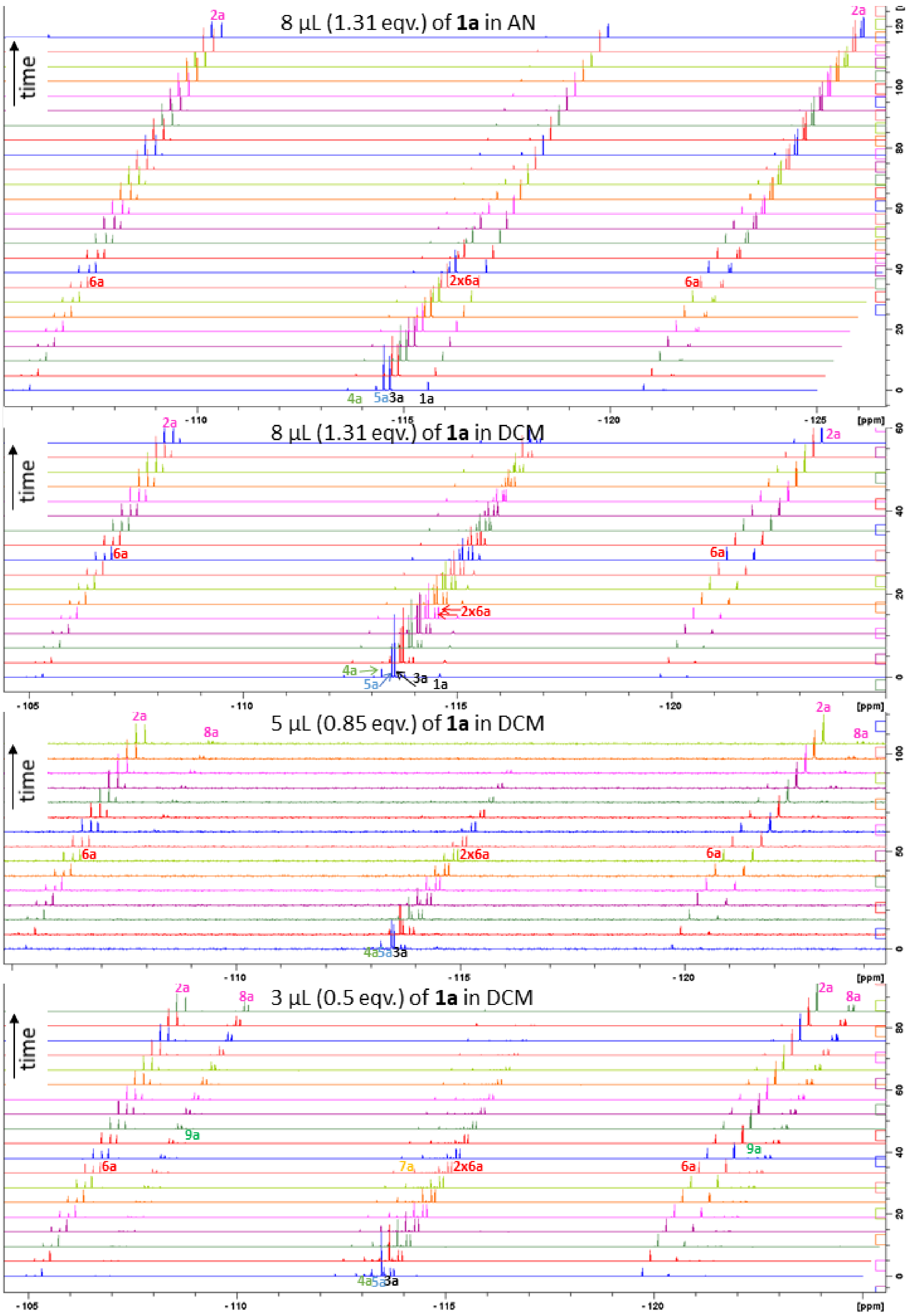

Figure S22. The ^{19}F NMR signal assignment for reaction of 8 various amounts of **1a** with 10 mg of 'Pd(OAc)$_2$' in acetonitrile (top) or CD_2Cl_2 (others) at room temperature.

Characterization of the compounds

4-fluoro-*N*,*N*-dimethylbenzylamine

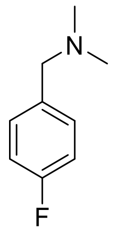

Appearance: yellow liquid

^{1}H NMR (500 MHz, $CDCl_3$): δ 7.26 (dd, ^{3}J 8.8 and 5.8 Hz, 2H); 6.99 (t, ^{3}J 8.73 Hz, 2H); 3.37 (s, 2H); 2.22 (s, 6H).

^{13}C{1H} NMR (100 MHz, $CDCl_3$): δ 162.0 (d, J = 244 Hz); 134.6 (d, J = 2.89 Hz);130.6 (d, J = 7.94 Hz); 115.0 (d, J = 21.36 Hz); 63.6; 45.3.

^{19}F{1H} NMR (376 MHz, $CDCl_3$): δ -115.97

The spectral data are in agreement with reference 2.

di-*μ*-acetato-bis-[*o*-dimethylaminomethyl-4-fluorophenyl-*C*,*N*) dipalladium(II) (2b)

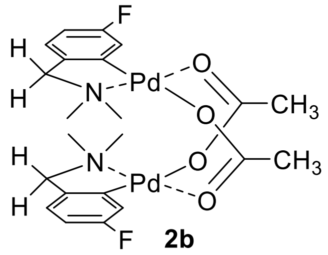

2b

Appearance: yellow solid

^{1}H NMR (400 MHz, $CDCl_3$): δ 6.81 (m, 2H,); 6.69 (m, 4H); 3.60 (d, ^{3}J 13.51 Hz, 2H); 3.14 (d, ^{3}J 13.51 Hz, 2H); 2.79 (s, 6H); 2.09 (s, 6H); 2.07 (s, 6H).

^{13}C{1H} NMR (100 MHz, $CDCl_3$): δ 181.2; 159.0 (d, J = 251.1 Hz); 146.4 (d, J = 8.1 Hz); 142.4 (d, J = 4.9 Hz); 121.9 (d, J = 7.4 Hz); 118.4 (d, J = 18.1 Hz); 110.9 (d, J = 22.9 Hz); 71.9; 52.4; 51.3; 24.5.

^{19}F{1H} NMR (376 MHz, $CDCl_3$): δ -115.9

FTIR (ATR): cm^{-1} 3052; 2983; 2929; 2843; 1598; 1575; 1463; 1413; 1345.

Crystallography

Table S4: Experimental details for **2b.8b**

Crystal data (CCDC 2189187)	
Chemical formula	$C_{26}H_{34}F_2N_2O_8Pd_3 \cdot 2(C_{22}H_{28}F_2N_2O_4Pd_2)$
M_r	2130.27
Crystal system, space group	Monoclinic, $P2_1/c$
Temperature (K)	150
a, b, c (Å)	21.3179 (11), 9.6634 (6), 19.6558 (12)
β (°)	102.780 (2)
V (Å^3)	3948.8 (4)
Z	2
Radiation type	Mo $K\alpha$
μ (mm^{-1})	1.64
Crystal size (mm)	0.24 × 0.18 × 0.12
**Data collection**	
Diffractometer	Bruker D8 - Venture
Absorption correction	Multi-scan *SADABS2016*/2 - Bruker AXS area detector scaling and absorption correction
T_{min}, T_{max}	0.591, 0.746
No. of measured, independent and observed [$I > 2\sigma(I)$] reflections	82575, 9117, 6799
R_{int}	0.210
$(\sin \theta/\lambda)_{max}$ (Å^{-1})	0.651
**Refinement**	
$R[F^2 > 2\sigma(F^2)]$, $wR(F^2)$, S	0.081, 0.147, 1.21
No. of reflections	9117
No. of parameters	486
H-atom treatment	H-atom parameters constrained
	$w = 1/[\sigma^2(F_o^2) + (0.0081P)^2 + 53.6466P]$ where $P = (F_o^2 + 2F_c^2)/3$
$\Delta\rho_{max}$, $\Delta\rho_{min}$ (e Å^{-3})	1.48, −1.67

Computer programs: Bruker Instrument Service vV6.2.3, *APEX3* v2016.9-0 (Bruker AXS), *SAINT* V8.37A (Bruker AXS Inc., 2015), *SHELXT* 2014/5 (Sheldrick, 2014), *SHELXL2017*/1 (Sheldrick, 2017), Bruker *SHELXTL*.

The X-ray data for colorless crystals of **2b.8b** were obtained at 150K using Oxford Cryostream low-temperature device with a Bruker D8-Venture diffractometer equipped with Mo (Mo/K_α radiation; λ = 0.71073 Å) microfocus X-ray (IμS) source, Photon CMOS detector and Oxford Cryosystems cooling device was used for data collection. Obtained data were treated by XT-version 2014/5 and SHELXL-2017/1 software implemented in APEX3 v2017.0-1 (Bruker AXS) system.[3] $R_{int} = \sum|F_o^2 - F_{o,mean}^2|/\sum F_o^2$, $S = [\sum(w(F_o^2 - F_c^2)^2)/(N_{diffrs} - N_{params})]^{½}$ for all data, $R(F) = \sum||F_o| - |F_c||/\sum|F_o|$ for observed data, $wR(F^2) = [\sum(w(F_o^2 - F_c^2)^2)/(\sum w(F_o^2)^2)]^{½}$ for all data. Crystallographic data for structural analysis have been deposited with the Cambridge Crystallographic Data Centre, CCDC no. 2189187. Copies of this information may be obtained free of charge from The Director, CCDC, 12 Union Road, Cambridge CB2 1EY, UK (fax: +44-1223-336033; e-mail: deposit@ccdc.cam.ac.uk or www: http://www.ccdc.cam.ac.uk).

The frames for all complexes were integrated with the Bruker SAINT software package using a narrow-frame algorithm. Data were corrected for absorption effects using the Multi-Scan method (SADABS). The structures were solved and refined using the Bruker SHELXTL Software Package.
Hydrogen atoms were mostly localized on a difference Fourier map, however to ensure uniformity of treatment of crystal, most of the hydrogen atoms were recalculated into idealized positions (riding model) and assigned temperature factors Hiso(H) = 1.2 Ueq (pivot atom) or of 1.5Ueq (methyl). H atoms in methyl, methylene moieties and C-H in aromatic rings were placed with C-H distances of 0.96, 0.97 and 0.93 Å. Artefacts were assigned to the Pd atoms with occupancy of ~6%.

Representative Spectral Data

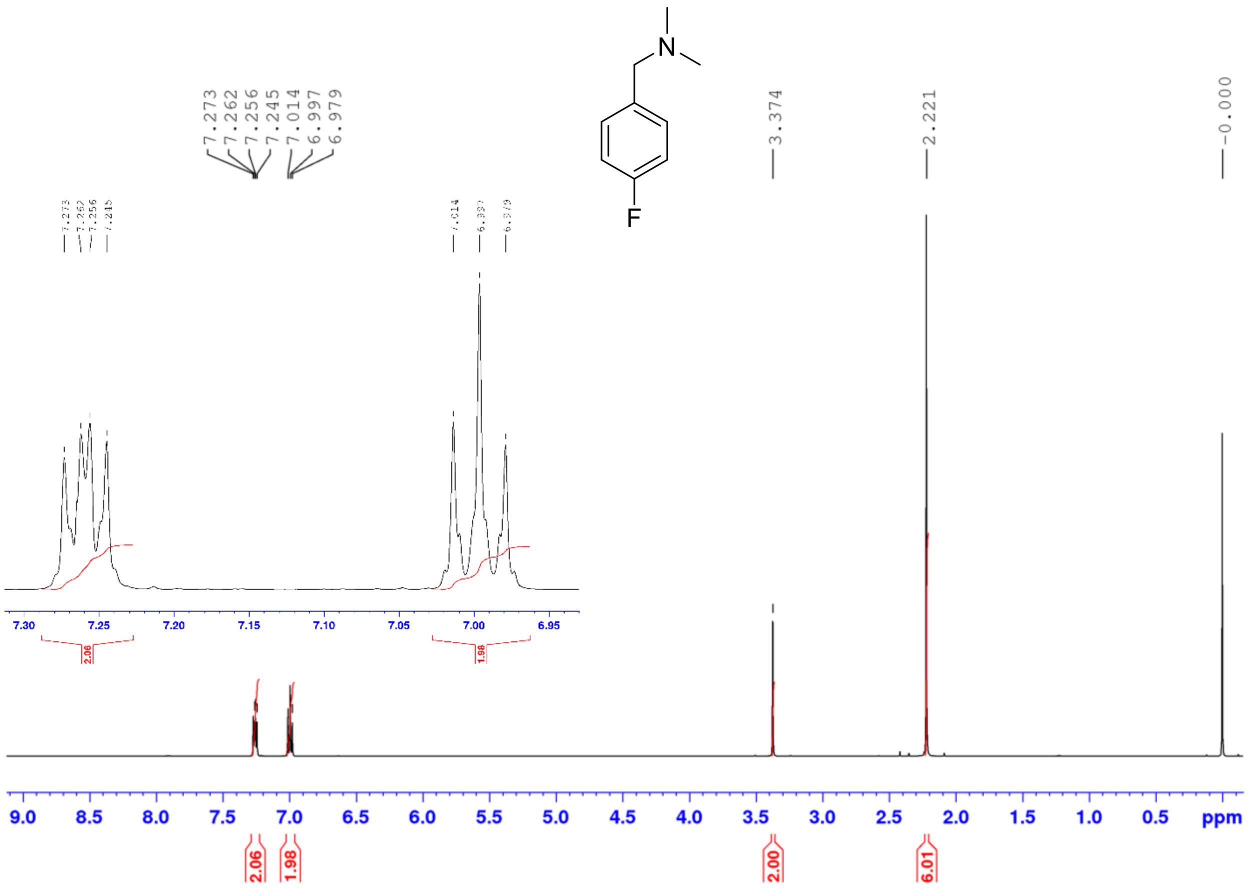

Figure S23. ^{1}H-NMR of 4-fluoro-*N*,*N*-dimethylbenzylamine (**1b**) at 298 K in $CDCl_3$ (500 MHz).

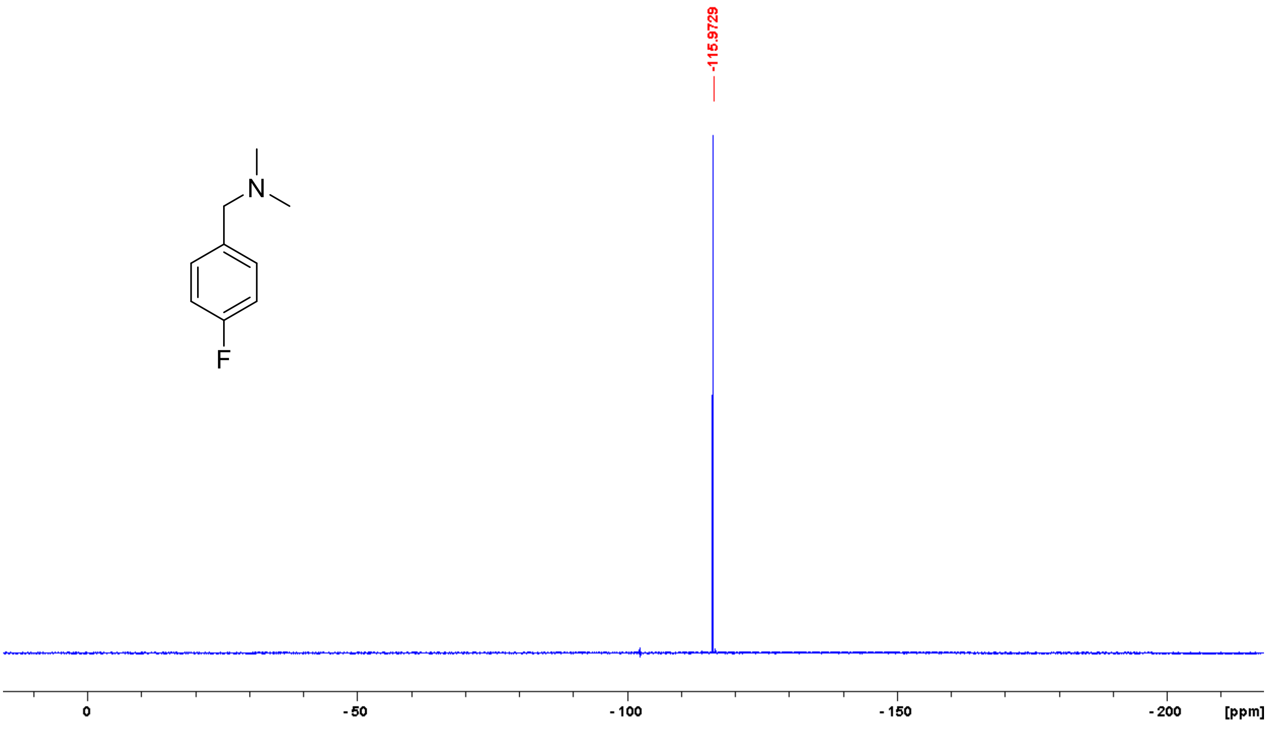

Figure S24. ^{19}F{H}-NMR of 4-fluoro-*N*,*N*-dimethylbenzylamine (**1b**) at 298 K in $CDCl_3$ (376 MHz).

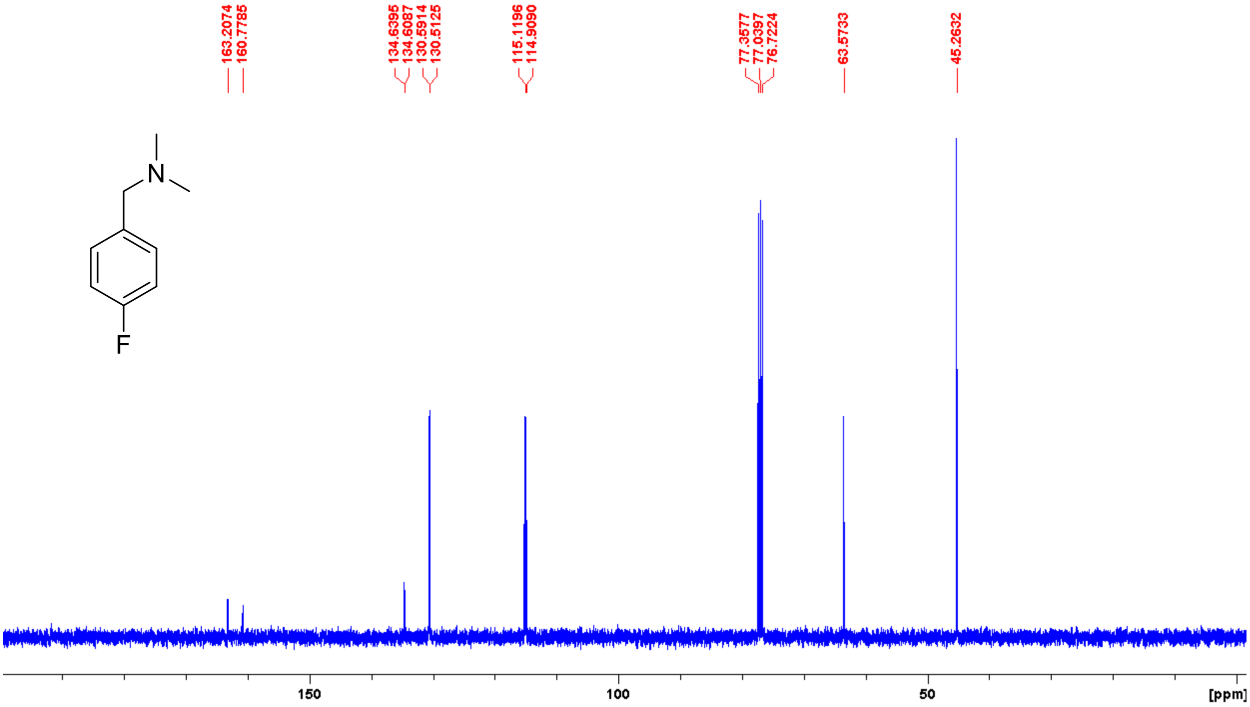

Figure S25. ^{13}C-NMR of 4-fluoro-*N*,*N*-dimethylbenzylamine (**1b**) at 298 K in $CDCl_3$ (100 MHz).

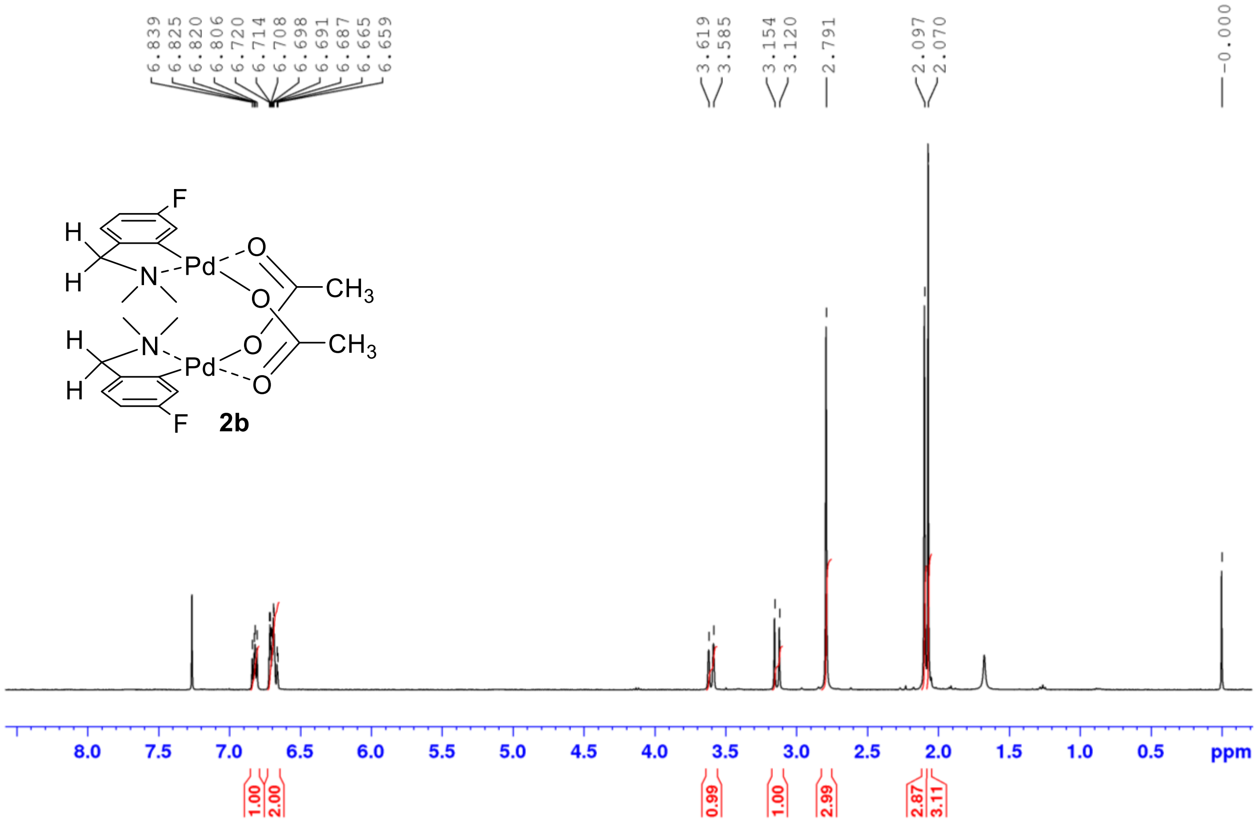

Figure S26. ^{1}H-NMR of di-*μ*-acetato-bis-[*o*-dimethylaminomethyl-4-fluorophenyl-*C*,*N*) dipalladium(II) (**2b**) at 298 K in $CDCl_3$ (400 MHz).

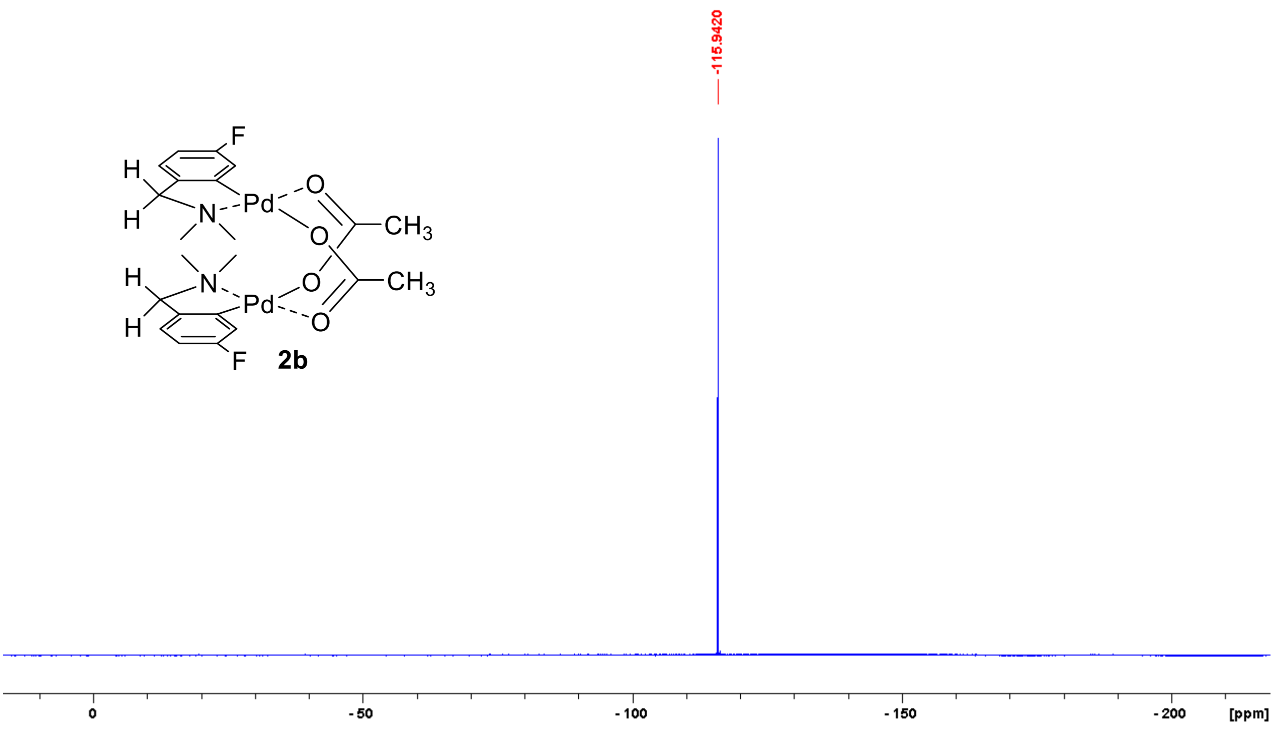

Figure S27. $^{19}F\{H\}$-NMR of di-μ-acetato-bis-[*o*-dimethylaminomethyl-4-fluorophenyl-*C*,*N*) dipalladium(II) (**2b**) at 298 K in $CDCl_3$ (376 MHz).

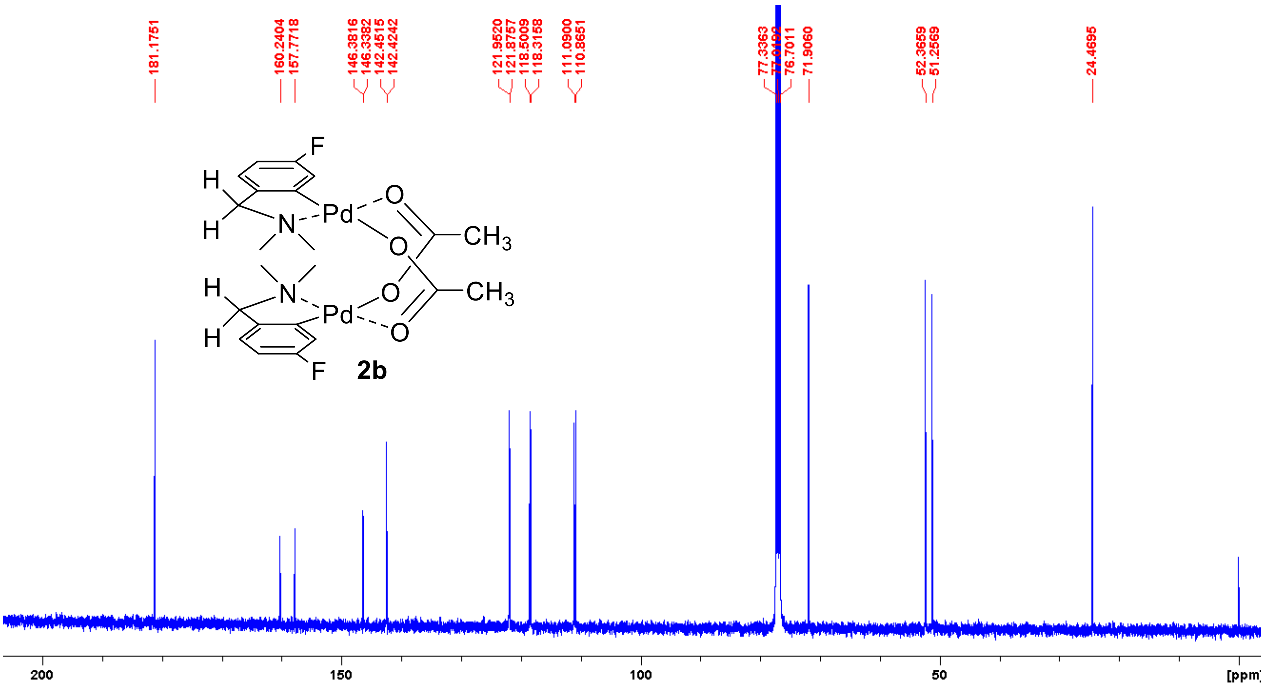

Figure S28. ^{13}C-NMR of di-μ-acetato-bis-[*o*-dimethylaminomethyl-4-fluorophenyl-*C*,*N*) dipalladium(II) (**2b**) at 298 K in $CDCl_3$ (100 MHz).

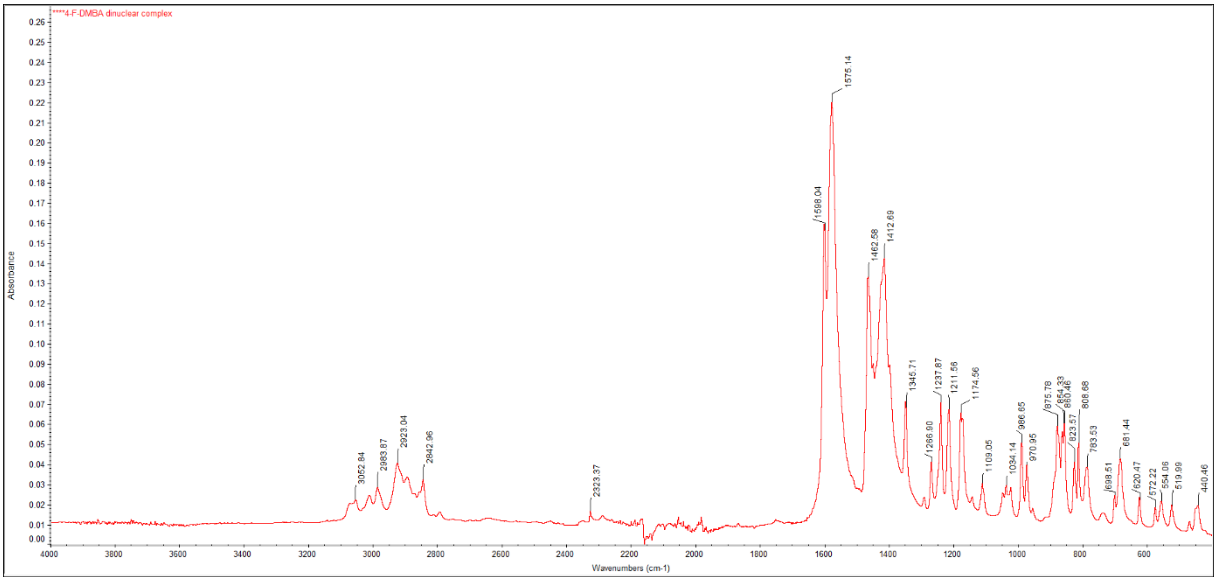

Figure S29. FTIR-ATR of spectrum of di-*μ*-acetato-bis-[*o*-dimethylaminomethyl-4-fluorophenyl-*C*,*N*) dipalladium(II) (**2b**).

References

1. The dielectric constants values are taken from: Solvents and Solvent Effects in Organic Chemistry, Fourth Edition, C. Reichardt, T. Welton, Wiley-VCH Verlag GmbH & Co. KGaA, First published **2010**, ISBN:9783527324736.

2. Feng, R.; Yao, J.; Liang, Z.; Liu, Z.; Zhang, Y. Cu(II)-Promoted Palladium-Catalyzed C–H Ortho-Arylation of *N*,*N*-Dimethylbenzylamines, *J. Org. Chem.* **2013**, *78*, 3688–3696.

3. Sheldrick, G. M. SHELXT-Integrated Space-Group and Crystal-Structure Determination. *Acta Cryst. A71* **2015**, 3-8.
